# Nanobodies Outperform Antibodies – Rapid Functionalization with Equal In Vivo Targeting Properties

**DOI:** 10.1002/adma.202412563

**Published:** 2024-10-29

**Authors:** Carina Jung, Michael Fichter, Jennifer Oberländer, Jenny Schunke, Vanessa Bolduan, Paul Schneider, Jinhong Kang, Kaloian Koynov, Volker Mailänder, Katharina Landfester

**Affiliations:** ^1^ Max Planck Institute for Polymer Research Ackermannweg 10 55128 Mainz Germany; ^2^ Department of Dermatology University Medical Center of the Johannes Gutenberg University Mainz Langenbeckstr. 1 55131 Mainz Germany

**Keywords:** dendritic cell targeting, nanobody, nanocarrier

## Abstract

Highly specific targeting of dendritic cells in vivo is crucial for the development of effective tumor nanovaccines. This group recently presented an antibody‐functionalized nanocarrier system able to maintain its targeting properties when transferred from in vitro to in vivo studies. However, producing this system requires long synthesis times and involves high expenses due to the involved site‐specific enzymatic multi‐step modification procedure of the antibody. Consequently, improving the previously proposed system is necessary in order to accelerate the development. Here, a novel system utilizing nanobodies for the targeting of dendritic cells is presented. A C‐terminal cysteine tag facilitates an easy attachment of the nanobody to the nanocarrier via a thiol‐maleimide conjugation technique. This reduces the functionalization time from several days to mere hours. Using in vitro and in vivo assays, it is shown that the optimized system possesses equal targeting properties as the antibody‐based system. As a result, nanobodies and the coupling chemistry are found to be a superior strategy for the in vivo targeting of dendritic cells when compared to antibodies, due to their rapid attachment to nanocarriers and equal targeting specificity. This would replace antibodies as the current “gold standard” of targeting moieties.

## Graphical abstract

A nanocarrier system functionalized with nanobodies for the targeting of dendritic cells is presented. The system possesses a highly specific targeting in vivo and can be synthesized at low cost over the course of a few hours. The combination of these properties suggests a better performance compared to antibody‐based systems in current literature.

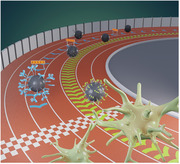

## 1 Introduction

Recently, with the development of therapeutical nanovaccines, the concept of addressing the immune system directly by vaccination has prompted a new high interest in the treatment of tumors via anti‐tumor‐nanovaccines. These nanovaccines – as immunotherapy in general – provide a means of avoiding the administration of large doses of harmful drugs and limiting the resulting side effects, which occur for example during chemotherapy.

In the development of effective immunotherapeutic nanovaccines, a major aim is to achieve a targeted and specific delivery of antigens to dendritic cells.^[^ ^1^ ^]^ This cell type is of great importance for the induction of antigen‐specific immune responses due to its ability to prime cytotoxic T cells following antigen uptake and presentation on the cell surface.^[^ ^2^ ^]^ Upon priming, cytotoxic T cells can recognize virus‐infected cells or cancer cells, subsequently leading to the elimination of these cells.^[^ ^3^ ^]^ Therefore, delivery of the nano vaccine directly – and specifically – to dendritic cells is crucial for high therapeutic success. For this purpose, targeting moieties addressing receptors exclusively expressed on dendritic cells, such as the CD11c receptor (CD: cluster of diffentiation), must be attached to the nanocarrier building the basis of the nanovaccine.

Antibodies (ABs) are the most commonly used targeting moiety described in the literature, however, their attachment to nanocarriers for targeting purposes by conventional methods bears the risk of random orientation of the antibody on the nanocarrier due to non‐site‐specific functionalization. In this case, the Fc domain (Fc: fragment crystallizable) of the antibody could protrude from the nanocarrier surface and enable unspecific binding via their non‐active Fc domain, leading to, for instance, uptake in macrophages.^[^ ^4^ ^]^ This issue of unspecific binding can be resolved by introducing a site‐specific enzymatic modification of the antibodies’ carbohydrate groups only present at the Fc domain, followed by a strain‐promoted azide‐alkyne click reaction to link the antibody to the corresponding nanocarrier.^[^ ^5^ ^]^ In this case, the benefit of click chemistry is twofold: First, it provides a quick reaction under mild conditions with a high reaction efficiency. Second, the inherent biorthogonality of the reaction, due to the non‐physiological nature of the involved functional groups, maintains the binding properties of the antibody by leaving the active regions untouched.^[^ ^6^ ^]^ Simultaneously, this attachment prevents an exposure of the non‐specifically binding Fc part on the nanocarrier surface. As an alternative, genetically engineered antibodies with Fc domains with lowered binding affinity to Fc receptors or complement proteins could be used, but the development of such antibodies can be tedious, making a chemical modification more desirable.^[^ ^7^ ^]^ The combination of biorthogonality and positioning of the antibody on the nanocarrier surface results in an excellent targeting of dendritic cells in vivo, while avoiding uptake by non‐dendritic cells like macrophages,^[^ ^8^ ^]^ a task that many proposed systems have failed to demonstrate in early in vivo studies.

However, while the attachment of the antibody by click chemistry is highly specific and straightforward, the obtained system is difficult to characterize, a prerequisite for subsequent translation into clinical studies. More importantly, the required multi‐step enzymatic modification of the antibody prior to its attachment is very cost‐intensive and time‐consuming, leaving much room for improvement. Here, we aim to provide this necessary improvement by designing and optimizing a system with equal targeting specificity toward dendritic cells and significantly improved synthesis conditions.

A promising approach is the replacement of antibodies with the antigen‐binding part of a cameloid antibody commonly referred to as nanobody (NB), which has been described for different nanoparticle classes, such as polymeric nanogels or polymersomes.^[^ ^9^ ^]^ As opposed to conventional antibodies, where the heavy and light chains would need to be linked together to form a nanobody, oftentimes leading to unstable constructs or a loss of the antigen‐binding function, the antigen‐binding part of this specific type of heavy‐chain‐only antibody consists of one amino acid chain only and can therefore even be produced in bacteria in high quantities. Several studies have described a low or negligible immunogenicity of the camelid‐derived nanobodies^[^ ^10^ ^]^ and humanization can be conducted in order to apply nanobodies in humans.^[^ ^11^ ^]^

Furthermore, nanobodies lack the Fc domain responsible for unspecific binding to non‐target cells, thereby circumventing the issue of a possibly lacking targeting specificity. Therefore, the advantage of CD11c‐binding nanobodies with a C‐terminal cysteine over antibodies is represented by the site‐specific attachment without prior modification,^[^ ^9^, ^12^ ^]^ reducing the overall synthesis time to hours, rather than days. The full process results in a noticeably lower production cost. As before, the implemented thiol‐maleimide reaction involved in the covalent attachment of our nanobody to the nanocarrier falls within the category of click reactions: It can be carried out in high yields and under mild reaction conditions, which preserves the biological activity of the protein.^[^ ^13^ ^]^

By combining this remarkable improvement of the production process with an exceptional targeting specificity, the surface functionalization of nanocarriers with nanobodies may open the opportunity for a new class of nanobody‐based immunotherapeutical nanovaccines for the ongoing fight against cancer.

## 2 Results and Discussion

As previously described by our group, a site‐selective modification of anti‐CD11c antibodies (ABs) with azide groups, followed by an attachment to nanocarriers via a dibenzocyclooctyne (DBCO) linker, leads to an effective targeting of dendritic cells in vitro and in vivo.^[^ ^14^ ^]^ While this method yields excellent results, it would be preferable to avoid the time‐consuming and costly azide modification step of the antibodies, which is necessary to achieve site‐selective attachment and prevent an unspecific Fc‐mediated uptake of the functionalized carriers into cells.

**Figure 1:**
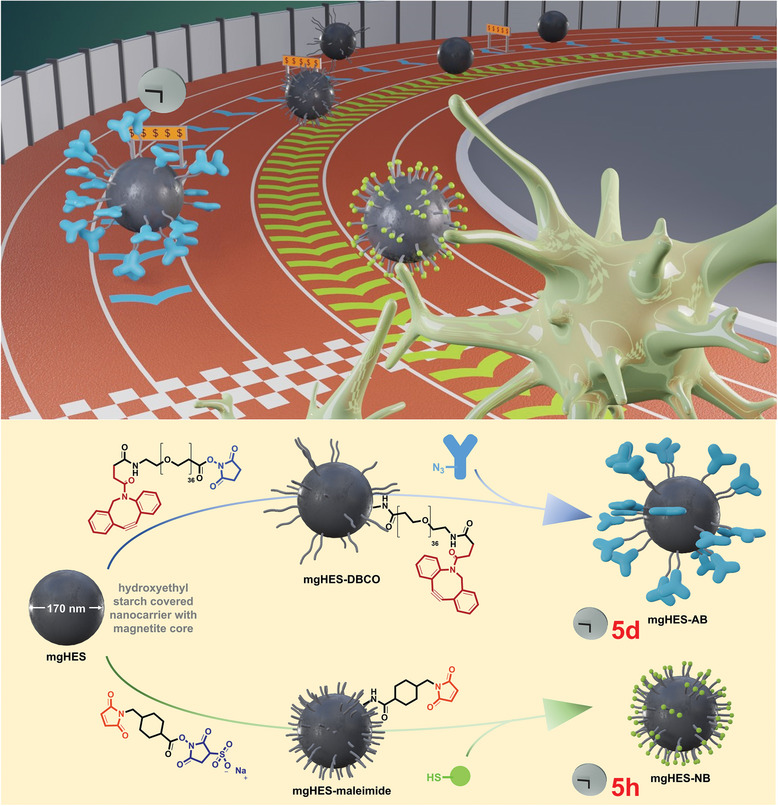
Concept overview. Replacement of the previously reported antibody‐based system with a nanobody‐based nanocarrier system leads to a significantly reduced functionalization time while maintaining targeting properties.

Nanobodies with a cysteine tag offer a promising alternative, as they only consist of the active region of an antibody. This means that no unspecific binding will occur. Simultaneously, the cysteine groups of our nanobodies allow for a rapid functionalization via maleimide‐thiol click reaction without prior modification of the nanobody, leading to a significant advantage when considering production time and cost (**Figure** **1**).

While the thiol‐maleimide reaction is a well‐established method for the coupling of proteins, its application to nanobodies in order to form a novel system with much‐improved synthesis conditions is very promising. In the following, we present such a nanobody‐conjugated nanocarrier system and demonstrate its rapid functionalization, as well as its high targeting specificity toward dendritic cells.

### 2.1 Synthesis and Physicochemical Characterization of Nanobody‐Functionalized Nanocarriers

Core‐shell nanocarriers with a magnetic core consisting of multiple magnetite nanoparticles and a surrounding hydroxyethyl starch shell (mgHES) form the base of the targeting system. On average, the nanocarriers have a hydrodynamic diameter of ≈170 nm according to dynamic light scattering (DLS) measurements (Figure S1, Supporting Information), with a thickness of the hydroxyethyl starch shell of roughly 11 nm (calculated from thermogravimetric analysis (TGA) results, Figure S2, Supporting Information), and possess an aspect ratio of 1.5 (Figure S3, Supporting Information). While the magnetic core offers the advantage of a simple purification process by magnetic separation, the hydroxyethyl starch provides a stealth effect and additionally carries a low number of amine groups (3 nmol mg^−1^, or ≈3000 amine groups per nanocarrier).

#### 2.1.1 Nanocarrier Functionalization

**Figure 2:**
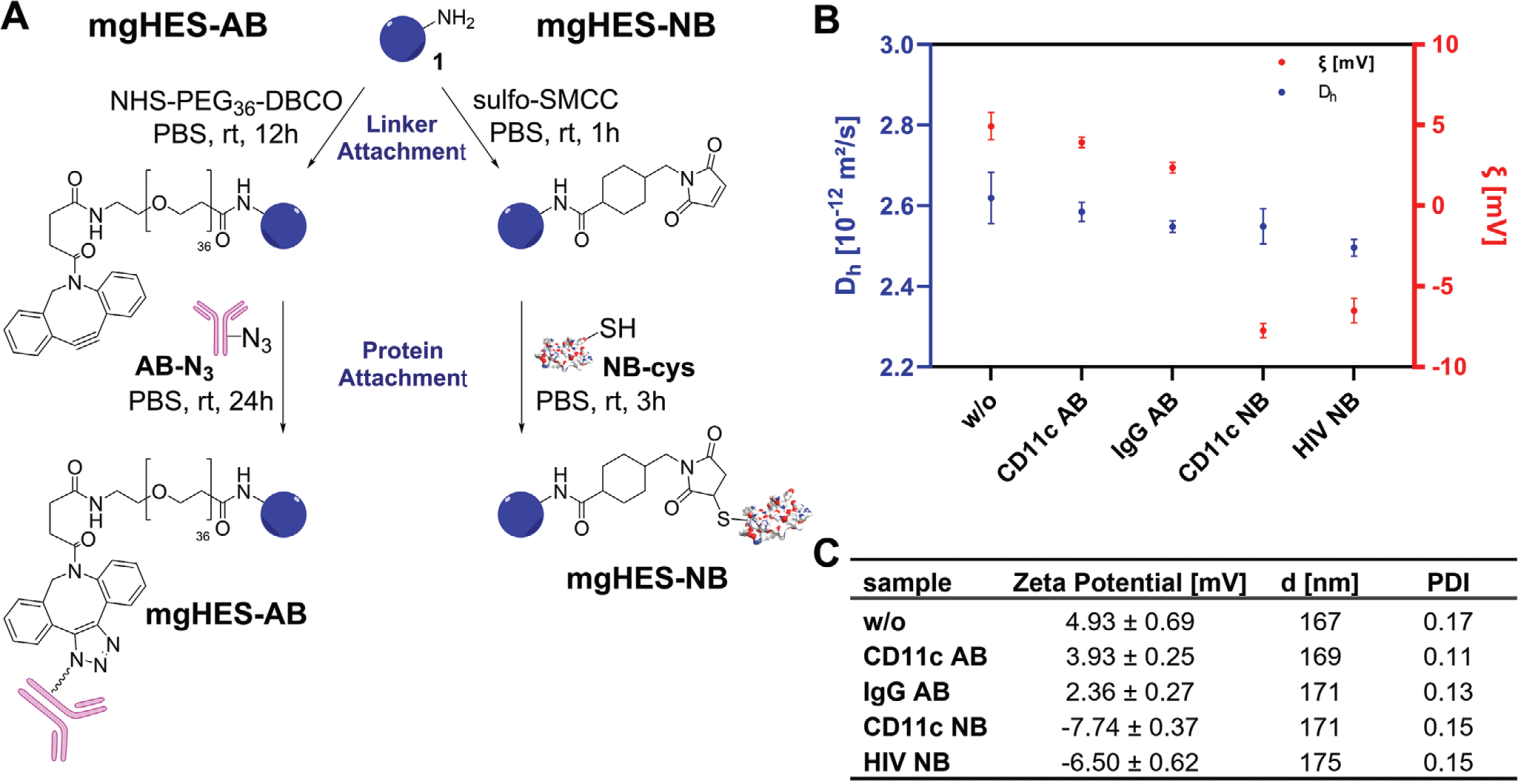
Synthesis and characterization of the nanocarrier‐protein conjugates. A) Covalent attachment of antibodies (AB, left) and nanobodies (NB, right) to magnetic nanocarriers (mgHES). B) Plotted diffusion coefficients and zeta potentials of unfunctionalized, antibody‐functionalized, as well as nanobody‐functionalized mgHES. Significantly lower zeta potentials for nanobodies compared to antibodies were observed. Samples with targeting antibody/nanobody and their corresponding control samples with IgG/HIV exhibited similar zeta potentials. C) Corresponding values, as obtained by measurement with Malvern Zetasizer in technical triplicates at pH 7.

In order to introduce clickable functional groups to the nanocarrier surface, these amine groups were used as anchor points for the attachment of linkers, which carry functional groups that allow for click reactions with proteins with compatible functionalities, as illustrated in **Figure** **2**A.

In the case of antibody‐functionalized mgHES (mgHES‐AB), a 2 kDa *N*‐hydroxysuccinimide‐poly(ethylene glycol)‐DBCO (NHS‐PEG_36_‐DBCO) linker was first attached to the carrier surface via its NHS moiety, followed by a reaction of the DBCO group with the azide‐functionalized antibody. The switch from a 5 kDa linker to a 2 kDa linker hereby leads to better uptake results (Figure S4, Supporting Information).^[^ ^15^ ^]^ Two antibodies were attached in this way, namely anti‐CD11c and its immunoglobulin G (IgG) isotype as a control. The azide functionalization was carried out over the course of three days in an enzymatic reaction in preparation for the attachment.^[^ ^5^ ^]^

In contrast, no prior modification of the nanobody was necessary, as it already contained a maleimide‐reactive C‐terminal cysteine tag, which could be used in a thiol‐maleimide coupling. This reaction is well‐established for the modification of proteins, but may be susceptible to hydrolysis over time, which would negatively impact the storage. However, the facile and rapid synthesis of the nanobody‐nanocarrier system (mgHES‐NB) allows for an on‐demand production of the targeting system, avoiding such complications.

For the coupling of the selected nanobody clone (selection according to binding affinity, see Figure S5, Supporting Information), its cysteine tag was exposed by reduction with tris(2‐carboxyethyl)phosphine hydrochloride (TCEP), making it accessible for further reactions. A sulfo‐succinimidyl‐4(N‐maleimidomethyl‐)cyclohexane‐1‐carboxylate (sulfo‐SMCC) linker was attached to the carrier surface via its NHS group, and the remaining maleimide functionality was combined with the nanobody in a second reaction step to form mgHES‐NB. A series of tests (Figures S6–S8, Supporting Information) revealed that this is not only a more rapid functionalization strategy but that this linking approach also leads to a more effective product when compared to a nanocarrier with nanobodies attached in a similar manner to the antibody, namely the DBCO‐azide strategy.

Analogous to the antibodies, a clone was selected that targets the CD11c receptor present on dendritic cells, as well as a negative control nanobody targeting the human immunodeficiency virus (HIV).

After functionalization, dynamic light scattering measurements (Figure 2B; Figure S1, Supporting Information) showed only minor differences in diffusion coefficient and, thus, size of the nanocarriers, suggesting that any changes in cell uptake behavior, later on, do not result from a change in size. Interestingly, while there was almost no change in zeta potential when attaching antibodies, the zeta potential values dropped by more than 10 mV when functionalizing with nanobodies (Figure 2B,C; Figure S9, Supporting Information). This may be a result of the added charge provided by the addition of the nanobodies and could lead to increased colloidal stability.

#### 2.1.2 Quantification of the Attached Nanobody Amount

The novel surface functionalization process of mgHES with nanobodies was investigated further in order to quantify the attached nanobody amount on the final conjugate. A fluorescamine assay revealed a reduction in the number of amine groups by about half in the linker attachment, suggesting a reaction efficiency of 50% for the first step at optimized conditions (10 eq. linker, see Figure S10, Supporting Information). The resulting maleimide surface functionalities (consequently ≈1.5 nmol mg,^−1^ or 1500 per nanocarrier) were then used for the nanobody attachment.

**Figure 3:**
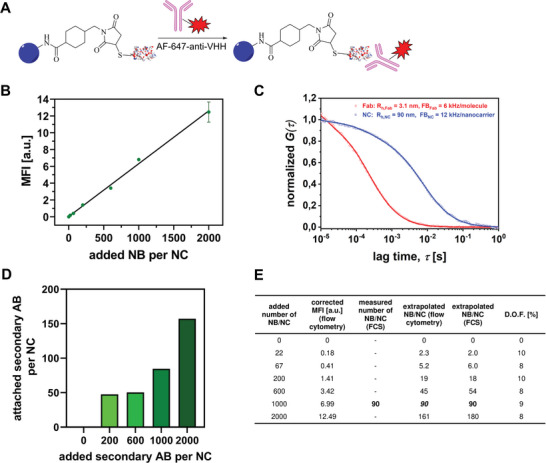
Investigation of the attached nanobody (NB) amount on the nanocarrier (NC) surface. A) Incubation with a nanobody‐binding Fab fragment of an IgG antibody (Fab: fragment antigen‐binding), or a whole secondary IgG antibody, carrying a fluorescence tag (AF647) allows for a qualitative and quantitative determination of the nanobody amount. B) Median fluorescence intensities (MFIs) of the mgHES‐NB samples after binding of the secondary antibody, as measured via nanoparticle‐only flow cytometric analyses in duplicates. A higher initial NB amount in the reaction mixture leads to a higher amount of NB on the NC surface after the completion of the reaction. The highest NB amount was additionally tested without the linker to confirm a covalent attachment and not an absorption effect. Additional data in SI (Figure S9, Supporting Information). C) Fluorescence Correlation Spectroscopy (FCS) investigations. Autocorrelation curves were measured for the 1000 NB/NC sample incubated with a large excess of a mixture of 2.2% dye‐tagged Fab in untagged Fab (blue) and dye‐tagged Fab only. The solid curves represent fits with Equation S1 (Supporting Information). D) Inverse binding experiment of a whole secondary IgG antibody carrying a fluorescence tag (AF647) to mgHES‐NB. E) Extrapolation of the attached nanobody amount using the MFIs determined by flow cytometry shows that the degree of functionalization is independent of the added nanobody amount, with 9 ± 1%.

As the nanobodies are very small, leading to no observable difference in electron microscopy (Figure S11, Supporting Information), a secondary antibody carrying a fluorescence tag was used to confirm the successful attachment of nanobodies to the carrier surface. Since this antibody binds to nanobodies specifically, the resulting fluorescence intensity per nanocarrier can be used to gain insight into the amount of attached nanobodies (**Figure** **3**A).

A qualitative investigation of the mgHES‐NB‐secondary antibody system was performed by particle‐only flow cytometry (Figure 3B). Here, a linear increase of the fluorescence intensity with an increasing amount of nanobody added in the initial reaction mixture was observed. When the nanobody was added under identical conditions, but without prior linker functionalization, there was no fluorescent signal (Figure S12, Supporting Information). This confirms that the nanobody does not absorb to the nanocarrier surface and must therefore be attached covalently.

Next, the exact number of nanobodies per nanocarrier was determined via fluorescence correlation spectroscopy (FCS, Figure 3C). In a typical FCS experiment, fluorescence intensity fluctuations caused by the diffusion of small fluorescent species such as dye molecules, labeled proteins, or nanocarriers through the “focus” of a confocal microscope are recorded. Autocorrelation‐based analysis of these fluctuations yields information on the hydrodynamic radius of the fluorescent species, their concentration, and fluorescent brightness (FB). Due to its high selectivity and single‐molecule sensitivity, FCS has become a powerful tool for the characterization of drug nanocarriers.^[^ ^16^ ^]^ Here, FCS experiments were performed with an exemplary mgHES sample with an added nanobody amount of 1000 per nanocarrier in the second reaction step. The NCs were mixed with a vast excess of Alexa Fluor 647 (AF647)‐labeled Fab fragments targeting the nanobodies, followed by incubation and magnetic purification to remove unbound Fab fragments. The Fab fragments were used instead of the antibodies to avoid the binding of one labeled antibody to two nanobodies at once with its two binding sites. Simultaneously, this ensures that possible grooves on the nanocarrier surface can be accessed due to the smaller size of the Fab fragment compared to the full secondary antibody. Furthermore, only 2.2 mol% of the Fab fragments incubated with the NCs were AF647‐labeled and the rest were unlabeled. This is a commonly used approach to avoid eventual fluorescence quenching due to a dense packing on the AF647 dyes on the NC surface.^[^ ^16^ ^]^

The typical FCS autocorrelation curve measured for this sample is shown (blue squares) in Figure 3C together with the autocorrelation curve measured for the solution of freely diffusing individual AF647‐labeled Fab fragments (red circles). Both curves were fitted with Equation S1 (Supporting Information), yielding the corresponding hydrodynamic radii and fluorescence brightness of the individual labeled Fab fragments and the NCs with attached Fab fragments. The hydrodynamic radius of the mgHES NCs is *R*_H,NC_ = 90 ± 5 nm which fits well with the value measured by DLS (Figure 2C). On the other hand, the fluorescent brightness of the NCs is FB_NC_ = 12 kHz/particle is two times larger than the one measured under identical conditions for the individual AF647‐labeled Fab fragments, FB_Fab_ = 6 kHz/molecule. This indicates that on average two AF647‐labeled Fab fragments were attached per NC. Considering that only 2.2 mol% of the Fab fragments were fluorescently labeled the FCS results show that for the exemplary mgHES sample with an initially added amount of 1000 nanobodies per nanocarrier, an amount of roughly 90 nanobodies per carrier was determined, which corresponds to a reaction efficiency of 9% in the second reaction step (Figure 3C,E).

By using this value for calibration of the mean fluorescence intensities determined via nanocarrier‐only flow cytometry for NCs with varying amounts of added nanobody per nanocarrier in the second reaction step (Figure 3A, second column in Figure 3E), the number of attached nanobodies per carrier was estimated for all samples (fourth column in Figure 3E). In all cases, a reaction efficiency of ≈9 ± 1% was calculated from the comparison of FCS and nanocarrier‐only flow cytometry, independent of the nanobody amount added in the second reaction step.

To further confirm these findings, an additional, indirect method was implemented. For this purpose, one equivalent of secondary antibody per equivalent of initially added nanobody was added to the purified sample. After incubation, the nanocarriers were removed by centrifugation, alongside any amount of bound secondary antibody, and the amount of antibody in the supernatant was determined by fluorescence calibration (Figure S13, Supporting Information). Here, a standard of the AF647‐tagged secondary antibody with a defined concentration was diluted repeatedly to generate further standards for a calibration curve, which was in turn used to calculate the concentration in the supernatant. The results for the mgHES samples with an added nanobody amount of 1000 per nanocarrier in the second reaction step nearly perfectly match the nanobody amount determined by FCS, with 85 attached antibodies compared to 90 attached Fab fragments. Furthermore, samples with 600 and 2000 nanobodies per nanocarrier were investigated in a similar manner. For all samples, ≈8% of the secondary antibody was bound to the nanocarriers and removed during centrifugation, suggesting a reaction efficiency of at least 8%.

By approximately calculating the surface area of the nanocarriers using the diameter and aspect ratio determined by DLS and TEM, the surface area per nanobody was determined. For the samples with an initial nanobody amount of 1000 per nanocarrier, ≈1000 nm^2^ per nanobody was calculated (Supporting Information).

The generous area available per nanobody suggests that the reaction efficiency of ≈10% is not a result of a densely packed nanobody functionalization, which would allow no further attachment, but must rather be caused by the inherent reactivity of the nanobodies and linker‐functionalized nanocarriers. Although the thiol‐maleimide reaction is often referred to as a click reaction due to its high yields, it may be limited by the size of the reactants and the accessibility of the reacting functional groups in the case of the nanocarrier‐nanobody conjugate. Similar findings have been described in the literature for the surface functionalization of nanocarriers. In fact, a functionalization degree of 10% seems to be quite high compared to yields achieved using other nanoparticle‐based systems.^[^ ^17^ ^]^

### 2.2 Biological Characterization

Following physicochemical characterization, the functionalized nanocarrier samples were evaluated using different cell lines, as well as an in vivo biodistribution assay, in which the nanobody sample exhibiting the highest cell uptake efficiency was compared to the previously published antibody functionalized sample. Additionally, a comparison of the protein corona of the antibody and nanobody functionalized samples was performed to ensure that any observed variations in uptake behavior are a result of targeting rather than protein corona composition.

#### 2.2.1 In Vitro Uptake of Nanobody‐Functionalized Carriers in Dendritic Cells

**Figure 4:**
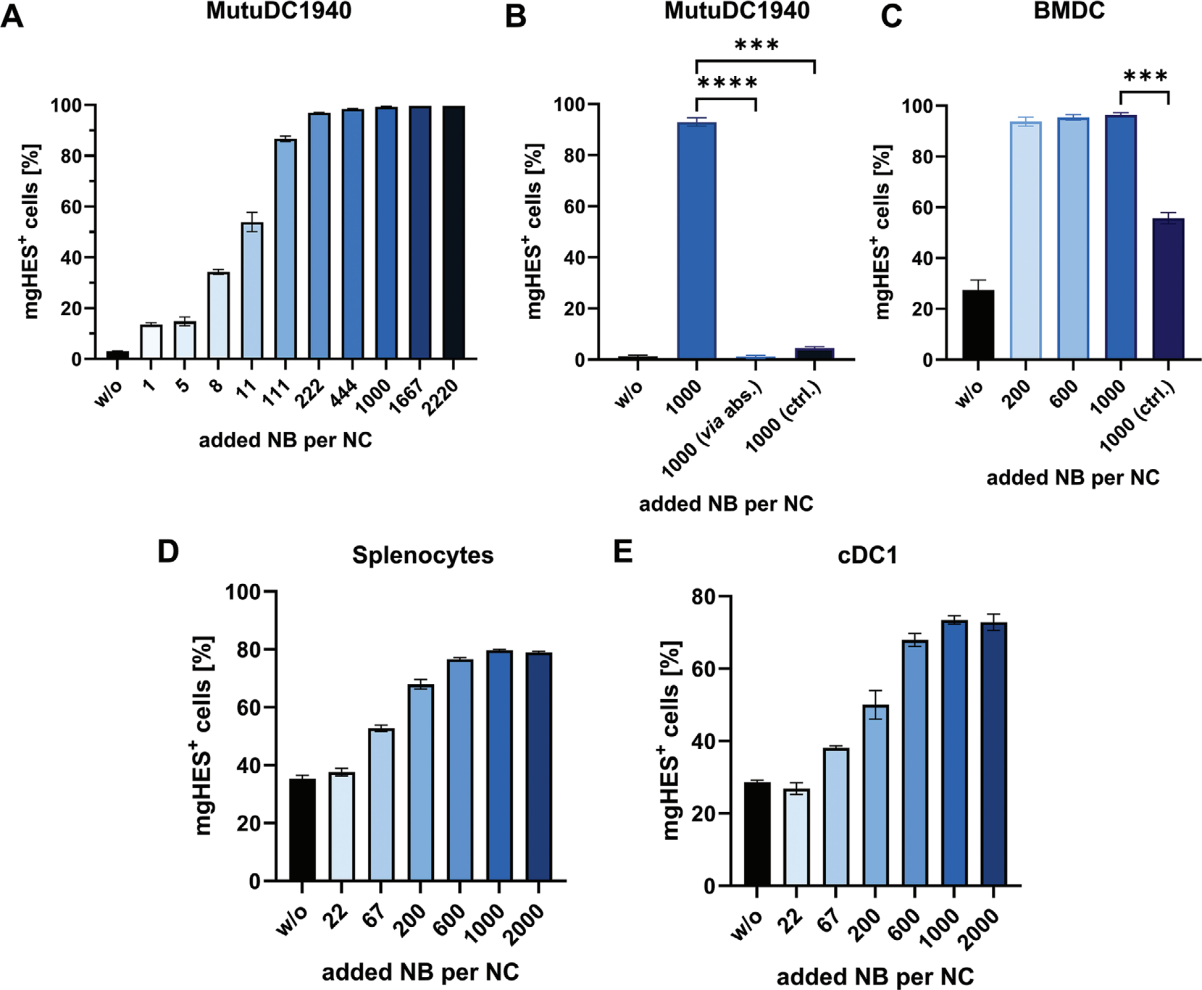
Determination of the optimal NB amount via in vitro studies with DCs. A) MutuDC1940 cells were incubated with mgHES nanocarriers functionalized with different amounts of targeting nanobodies for 2 h and uptake was analyzed using flow cytometry. B) Uptake by MutuDC1940 cells of covalently attached CD11c nanobody was compared to nanobodies absorbed to the mgHES surface (termed “via abs.”) and a control isotype (termed “ctrl.”). C–E) Nanocarriers with increasing amounts of CD11c nanobodies were incubated with either BMDCs (C) or splenocytes (D) for 20 h and uptake was analyzed using flow cytometry. E) Conventional DCs type 1 within splenocytes. Data represent the mean frequency (± SD) of nanocarrier‐positive cells (N = 3) and significance was given with *p* < 0.001^***^, *p* < 0.0001^****^ (One‐Way ANOVA).

To determine the optimal nanobody amount per nanocarrier (added NB per NC), nanocarriers functionalized with increasing amounts of nanobodies were tested in a series of in vitro assays regarding uptake by dendritic cells (DCs) (**Figure** **4**, additional data in Figures S14 and S15, Supporting Information).

A first trial in MutuDC1940, an immortalized cell line of conventional DCs type 1/cCD1), showed a saturation of the cells with the nanocarriers with increasing amounts of nanobody per nanocarrier (Figure 4A). For an added amount of 1000 nanobodies per carrier and higher, 100% of cells had taken up the nanocarriers. Additional uptake experiments for a sample modified with a reference nanobody, not binding to any receptor on dendritic cells, as well as a control sample with the nanobody absorbed to the nanocarrier, as opposed to covalently linked, and an identical purification protocol to the normal synthesis were performed as well (Figure 4B). Neither of the control samples showed a significantly higher uptake compared to the unmodified nanocarrier. This finding indicates on the one hand that the targeting is specific toward the CD11c receptor and not caused by the general presence of protein on the nanocarrier surface and, on the other hand, that the nanobodies are indeed covalently attached and do not absorb to the carrier surface.

A second trial with bone marrow‐derived dendritic cells (BMDCs) revealed a saturation of the cells at even lower amounts of nanobodies coupled to the nanocarriers (Figure 4C). However, the overall trends were identical to MutuDC1940 cells, with the sample with an initially added amount of 1000 nanobodies per nanocarrier reaching a saturation of close to 100% of nanocarrier‐positive cells.

Lastly, primary splenocytes were used to investigate the targeting capacity of nanobody‐functionalized nanocarriers in vitro (Figure 4D,E). Again, an increased uptake could be observed with increasing amounts of nanobodies. In this case, however, 100% saturation of the cells was not achieved. Instead, the sample with 1000 added nanobodies per carrier, which had resulted in full saturation in the other two cell types, now emerged as the superior sample with the highest uptake (percentage of ≈80%, see Figure S16, Supporting Information). This is especially well visible in cDC1 within the whole splenocyte population. cDC1 is a cell type very suitable for targeting due to its ability to cross‐present exogenous antigens to CD8^+^ T cells, which in turn can eliminate tumor cells after priming and activation by DCs.^[^ ^18^ ^]^ This mechanism is of great importance for the development of anti‐tumor vaccines.^[^ ^19^ ^]^

Since splenocytes are the closest in vitro equivalent of the in vivo behavior, the 1000 NB/NC sample was determined to be the optimum sample for further experiments.

#### 2.2.2 In Vitro Comparison of Antibody‐ and Nanobody‐Functionalized Nanocarriers

**Figure 5:**
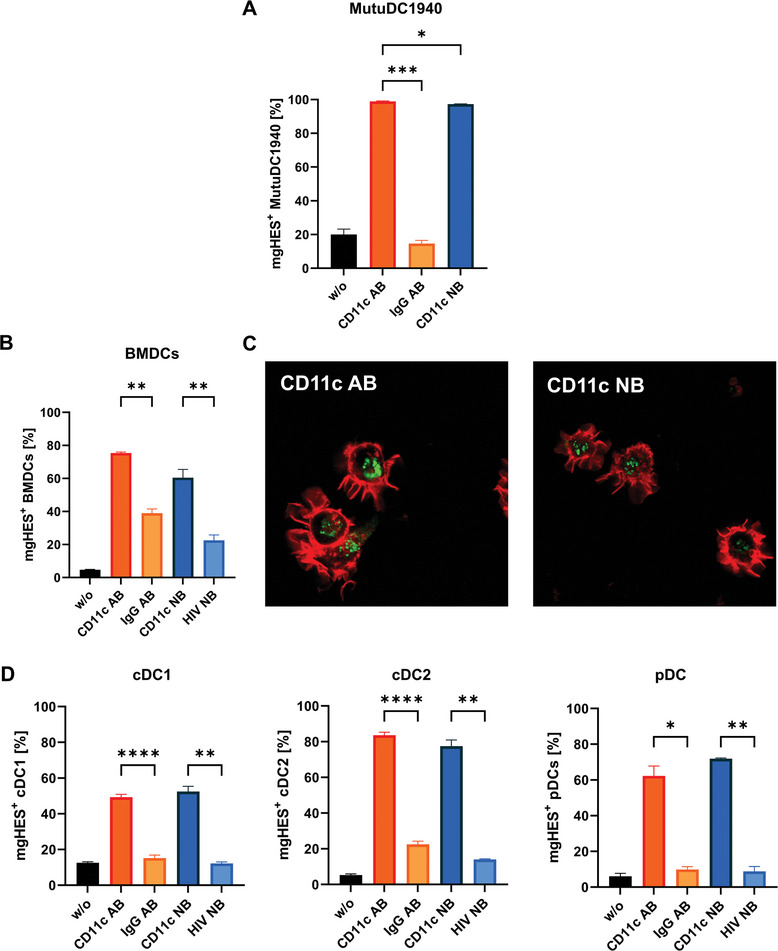
Uptake of mgHES‐AB and mgHES‐NB in different DCs in vitro. A) MutuDC1940 cells were incubated with antibody‐ or nanobody‐functionalized mgHES nanocarriers (30 µg mL^−1^) for 2 h. B) BMDCs were incubated with different nanocarrier formulations (30 µg mL^−1^) for 20 h. C) Confocal lases scanning microscopy (cLSM) images of BMDCs after 24 h incubation with 30 µg mL^−1^ of the antibody‐ or nanobody‐modified nanocarrier, respectively. Cell membranes were stained using CellMask DeepRed plasma membrane stain shortly before measurement. D) Splenocytes were incubated with different formulations of nanocarriers (30 µg mL^−1^) for 20 h and conventional DCs type 1, conventional DCs type 2, and plasmacytoid DCs were distinguished using flow cytometry. Data represent the mean frequency (± SD) of nanocarrier‐positive cells (N = 3) and significance was given with *p* < 0.05^*^, *p* < 0.01^**^, *p* < 0.001^***^, *p* < 0.0001^****^ (One‐Way ANOVA).

To compare the performance of nanobody‐ and antibody‐based nanocarriers, the optimized mgHES‐NB sample with 1000 initially added nanobodies per nanocarrier and the optimized mgHES‐AB sample^[^ ^5^, ^14^, ^15^ ^]^ were submitted to a second series of in vitro tests (**Figure** **5**). As before, the experiment was carried out in MutuDC1940, BMDCs, and splenocytes.

In MutuDC1940, a saturation of nearly 100% mgHES^+^ cells was reached after only two hours of incubation, with the nanobody sample leading to an overall higher median fluorescence intensity compared to the antibody sample (Figure S17, Supporting Information). This means that not only a higher percentage of cells take up the nanobody sample, but that the amount taken up per cell is higher as well compared to the antibody sample.

Lower frequencies of mgHES^+^ cells were achieved for BMDCs and splenocytes. In BMDCs, a higher, but not significant, uptake of mgHES‐AB than mgHES‐NB was observed. However, the MFI values achieved with the nanobody‐functionalized nanocarriers appeared to be significantly lower than the antibody‐functionalized nanocarriers (see Figure S17, Supporting Information).

In addition to the flow cytometric evaluation, the uptake of both samples targeted toward the CD11c receptor into BMDCs (Figure 5C; Figure S18, Supporting Information) and DC2.4 cells (Figure S19, Supporting Information) was observed using confocal laser scanning microscopy (cLSM). This method was implemented to prove that the fluorescent signal observed in flow cytometry stems from nanocarriers that reside inside the cells, rather than attaching to the cell surface. The corresponding images show that the nanocarriers are, in fact, located inside the cells, confirming that an uptake into the cells has taken place. Furthermore, the arrangement of the nanocarriers into round shapes suggests that they reside within the endosome.

Flow cytometric analyses of splenocytes (Figure 5D) showed a slightly higher uptake of the mgHES‐NB sample in cDC1 and plasmacytoid dendritic cells (pDCs) compared to the mgHES‐AB sample, and a slightly lower uptake in conventional dendritic cells type 2 (cDC2).

In all cell types, little to no uptake of the control samples with IgG and HIV‐NB was observed, confirming that the higher uptake is not a result of unspecific binding.

The contrary results in uptake with respect to the analyzed MFI values (see Supporting Information) achieved in MutuDC1940 cells and BMDCs can be explained by the different origins of both cell types since MutuDC1940 is a cDC1 cell line derived from a tumorigenic murine spleen and BMDC are cytokine‐derived inflammatory DCs generated from bone marrow cells *ex vivo*. In addition, CD11b^+^ macrophages within splenocytes were analyzed for their uptake of mgHES nanocarrier formulations, revealing a slightly higher uptake by functionalization with CD11c antibody (Figure S17, Supporting Information) that indicates a minor influence of Fc‐mediated recognition and internalization.

Since biodistribution and cell uptake of nanocarriers in the body cannot be precisely predicted from in vitro assays, an extensive in vivo characterization was performed (see Section 2.2.3).

#### 2.2.3 In Vivo Comparison of Antibody and Nanobody‐Functionalized Nanocarriers

**Figure 6:**
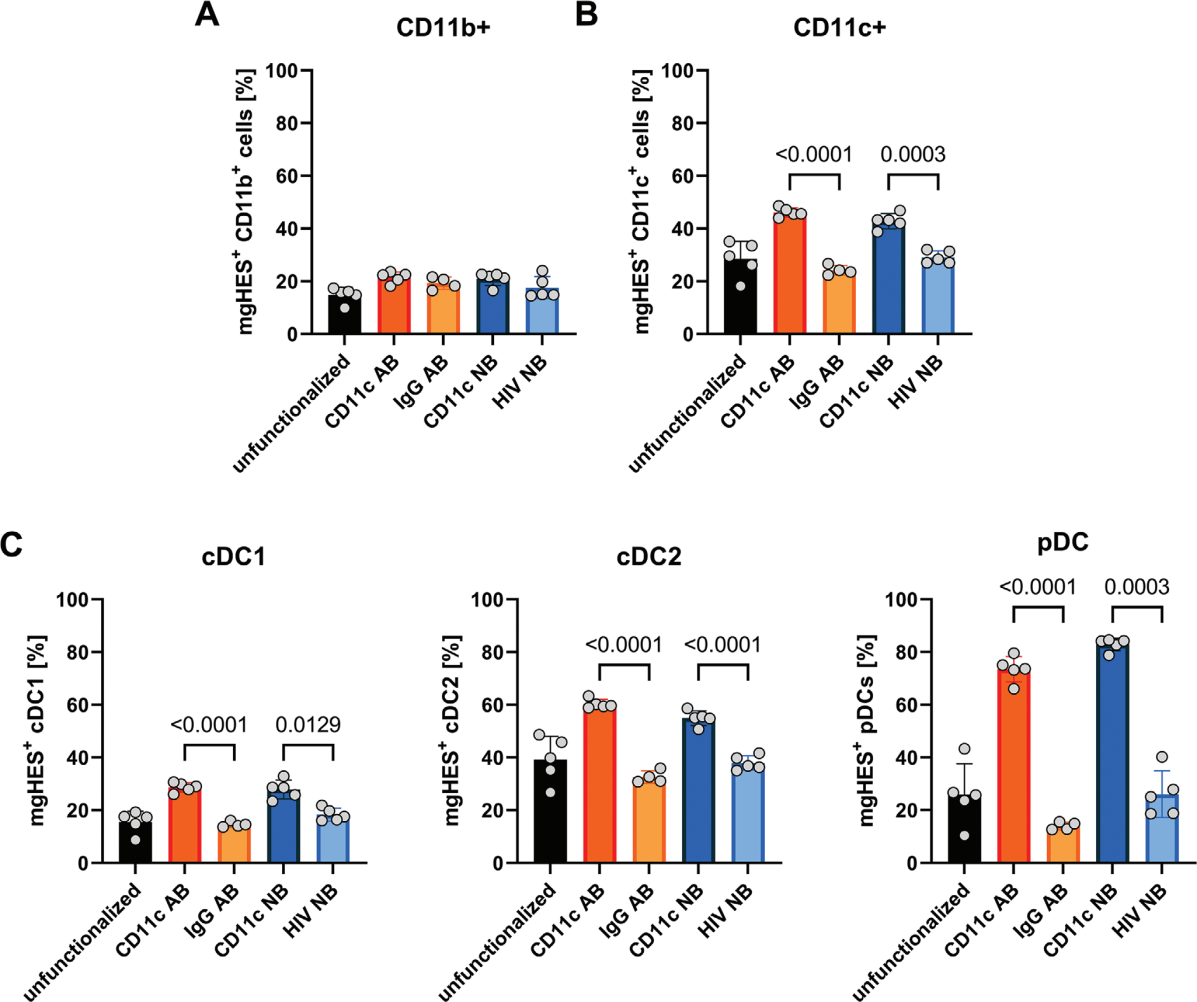
Targeting of dendritic cell subsets in vivo. mgHES nanocarriers functionalized with antibodies or nanobodies were intravenously injected into mice. Nanocarrier uptake by A) CD11c^+^ DCs, B) CD11b^+^CD11c^−^ macrophages, and C) DC subtypes (conventional DCs type 1, conventional DCs type 2, and plasmacytoid DCs) within the spleen was determined using flow cytometry. Data represent mean ± SD. CD11c antibody‐functionalized mgHES were compared to IgG control antibody‐functionalized mgHES and CD11c nanobody‐functionalized mgHES were compared to control nanobody (HIV) nanocarriers. Significance was given with *p* < 0.05 using a One‐Way ANOVA test. Individual *p* values are indicated in the graphs.

Numerous studies have shown selective targeting of antibody‐functionalized nanocarriers toward dendritic cells in vitro,^[^ ^20^ ^]^ however, many of them failed to retain this selectivity when applied in vivo.^[^ ^21^ ^]^ To show that the presented mgHES‐nanobody system can be successfully used for in vivo targeting, antibody‐ and nanobody‐functionalized mgHES nanocarriers were intravenously injected into mice and the uptake by different DC subtypes within the spleen was analyzed (**Figure** **6**). Subtype cDC1 represents the most important DC subtype for nanocarrier‐based anti‐tumor vaccination approaches due to their potential to efficiently cross‐present exogenous antigens to cytotoxic CD8^+^ T cells, which in turn leads to the elimination of tumor cells.^[^ ^22^ ^]^ IgG‐ and HIV‐nanobody‐functionalized nanocarriers served as respective negative controls for the CD11c antibody and nanobody nanocarriers. While no differences between all tested formulations could be observed when analyzing non‐targeted CD11b^+^ macrophages (Figure 6A), targeted CD11c^+^ DCs showed an increased uptake for both the CD11c‐antibody and CD11c‐nanobody nanocarriers (Figure 6B). This result is in accordance with our previously published study.^[^ ^8a^ ^]^ Similar findings were achieved when analyzing the DC subsets cDC1 (Figure 6C), cDC2 (Figure 6C) and pDCs (Figure 6C). For all analyzed DC subsets, uptake of antibody and nanobody‐functionalized nanocarriers was equally effective, while both formulations proved to be significantly superior compared to the respective negative controls (IgG AB and HIV NB). These findings indicate an optimal and highly specific targeting of DCs for both targeting systems.

Additionally, fluorescence in vivo imaging was performed to evaluate the biodistribution of mgHES formulations on the organ level (Figure S20, Supporting Information). For this purpose, unfunctionalized or functionalized mgHES nanocarriers were intravenously injected, mice were sacrificed 24 h post‐injection and organs were dissected. Heart, lung, liver, kidneys, spleen, and inguinal lymph nodes were analyzed for nanocarrier accumulation using small animal fluorescence imaging. No significant differences between the injected nanocarrier formulations could be observed for all investigated organs.

The potential toxicity of mgHES formulations was evaluated in vitro as well as in vivo by staining with Live/Dead Aqua and subsequent flow cytometric analysis (Figure S21, Supporting Information). In vitro cultivated splenocytes (Figure S22A, Supporting Information) and BMDCs (Figure S22B, Supporting Information) as well as splenocytes isolated 24 h after nanocarrier injection (Figure S22C, Supporting Information) did not show a decreased frequency of live cells compared to untreated cells or injection of PBS, indicating that all mgHES formulations were well tolerated.

#### 2.2.4 Ex Vivo Protein Corona

**Figure 7:**
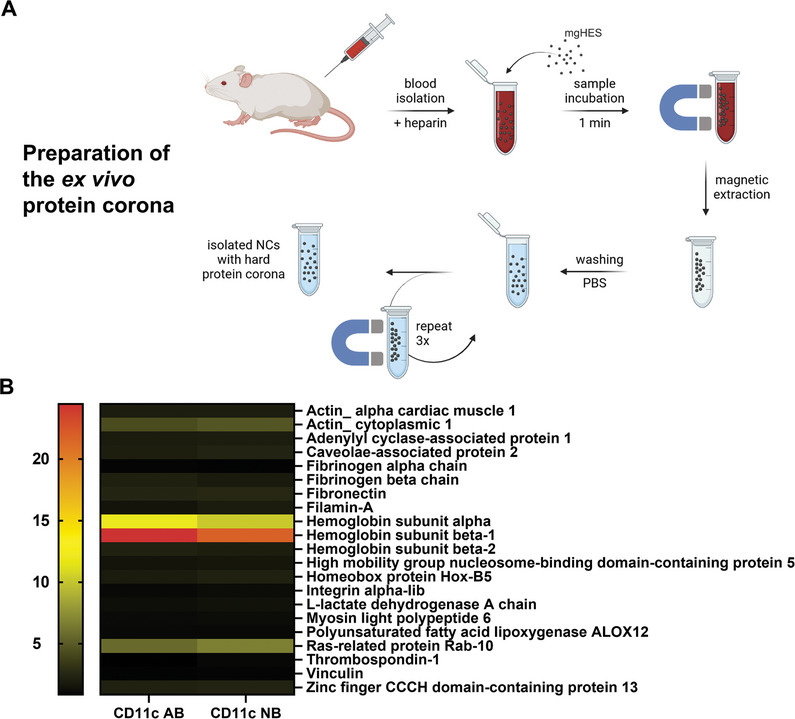
Ex vivo protein corona of mgHES‐NB and mgHES‐AB. A) Preparation process of the ex vivo protein corona by incubation of the NCs in murine blood (created in BioRender. Jung, C. (2023) BioRender.com/v27w055). B) Overview of the 20 most abundant proteins and their relative abundance in the protein corona.

In addition to the in vitro and in vivo uptake behavior of the developed nanocarrier systems, the *ex vivo* protein corona^[^ ^23^ ^]^ was characterized for the antibody and nanobody samples. For this purpose, the nanocarriers were incubated in anti‐coagulated murine full blood, immobilized by a magnet, washed in order to remove the loosely bound soft protein corona, and treated with an SDS‐based solution to desorb the hard protein corona from the nanocarriers. The hard corona proteins were separated from the nanocarriers by centrifugation and analyzed by liquid chromatography‐mass spectrometry (LC‐MS). The preparation process and the twenty most abundant proteins, as well as their relative frequencies, for the two nanocarrier samples, are depicted in **Figure** **7**.

Due to the use of whole blood, the most abundant proteins found in the protein corona for both nanocarriers are hemoglobin subunits (alpha and beta‐1), which are presented as the most abundant fraction in the blood control as well (Figure S22, Supporting Information). Furthermore, the amount of hemoglobin is likely additionally increased by the interaction of the magnetic core with the iron content of hemoglobin.

Besides fibronectin (acute phase protein), the fibrinogen chains (coagulation proteins), and L‐lactate dehydrogenase A chain (other plasma components), the remaining identified proteins are not typical blood components but rather can be seen as proteins originating from ´tissue leakage´ (e.g., actin cytoplasmic 1 and Ras‐related protein Rab‐10).^[^ ^24^ ^]^

Both proteins promoting an uptake into immune cells (opsonins), such as immunoglobulins and complement proteins, as well as stealth proteins (dysopsonins) like clusterin (ApoJ)^[^ ^25^ ^]^ are missing from the corona for both formulations tested.

The relative amount of serum albumin and hemoglobin subunit alpha were reduced in the protein corona of the nanocarriers in comparison to the composition of proteins for pure blood. Nevertheless, all in all, the profiles for the protein coronas of the CD11c antibody and nanobody‐modified samples are very similar (Figure 7B), suggesting that any differences in cell uptake are not a result of the protein corona.

## 3 Conclusion

In this study, we have presented a novel nanobody‐nanocarrier system defined by its rapid and cost‐effective synthesis. We have shown that, despite its much simpler production, the system possesses an equal targeting specificity toward dendritic cells in vivo when compared to a site‐specifically conjugated antibody‐nanocarrier system previously presented by our group. Furthermore, we have proven that an exact quantification of the attached nanobody amount on the carrier surface is possible via fluorescence correlation spectroscopy. This quantification will facilitate a translation of future nanovaccines with such a nanobody functionalization into clinical application. Consequently, due to their combined properties of a simple synthesis, an exact quantification, and an excellent selectivity toward dendritic cells in vivo, we expect nanobodies to replace antibodies as the most commonly used targeting moieties and become the new “gold standard” of targeting in the field of cancer immunotherapy. The translation of the herein presented nanobody functionalization of model nanocarriers to nanoparticles carrying antigens and adjuvants bears the potential to enhance anti‐cancer vaccine therapy in clinical settings and improve patient outcomes.

## 4 Experimental Section

Please refer to Supporting Information for experimental details.

## Conflict of Interest

The authors declare no conflict of interest.

## Supporting information



Supporting Information

## Data Availability

The data that support the findings of this study are available from the corresponding author upon reasonable request.

## References

[1] A. V. Baldin , L. V. Savvateeva , A. V. Bazhin , A. A. Zamyatnin, Jr. , Cancers (Basel) 2020, 12. 32150821 10.3390/cancers12030590 PMC7139354

[2] a) R. M. Steinman , Annu. Rev. Immunol. 1991, 9, 271; 1910679 10.1146/annurev.iy.09.040191.001415

[3] H. Raskov , A. Orhan , J. P. Christensen , I. Gögenur , Brit J Cancer 2021, 124, 359. 32929195 10.1038/s41416-020-01048-4 PMC7853123

[4] a) C. Kappel , C. Seidl , C. Medina‐Montano , M. Schinnerer , I. Alberg , C. Leps , J. Sohl , A. K. Hartmann , M. Fichter , M. Kuske , J. Schunke , G. Kuhn , I. Tubbe , D. Passlick , D. Hobernik , R. Bent , K. Haas , E. Montermann , K. Walzer , M. Diken , M. Schmidt , R. Zentel , L. Nuhn , H. Schild , S. Tenzer , V. Mailander , M. Barz , M. Bros , S. Grabbe , ACS Nano 2021, 15, 15191; 34431291 10.1021/acsnano.1c05713

[5] M. Brückner , J. Simon , K. Landfester , V. Mailänder , Nanoscale 2021, 13, 9816. 34031680 10.1039/d0nr08191d

[6] a) E. M. Sletten , C. R. Bertozzi , Angew. Chem., Int. Ed. 2009, 48, 6974; 10.1002/anie.200900942 PMC2864149 19714693

[7] a) T. H. Kang , S. T. Jung , Exp. Mol. Med. 2019, 51, 138; 31735912 10.1038/s12276-019-0345-9 PMC6859160

[8] a) J. Simon , M. Fichter , G. Kuhn , M. Brueckner , C. Kappel , J. Schunke , T. Klaus , S. Grabbe , K. Landfester , V. Mailaender , Nano Today 2022, 43;

[9] a) M. F. Debets , W. P. J. Leenders , K. Verrijp , M. Zonjee , S. A. Meeuwissen , I. Otte‐Höller , J. C. M. van Hest , Macromol. Biosci. 2013, 13, 938; 23695978 10.1002/mabi.201300039

[10] a) V. Cortez‐Retamozo , N. Backmann , P. D. Senter , U. Wernery , P. De Baetselier , S. Muyldermans , H. Revets , Cancer Res. 2004, 64, 2853; 15087403 10.1158/0008-5472.can-03-3935

[11] M. A. Rossotti , K. Belanger , K. A. Henry , J. Tanha , FEBS J. 2022, 289, 4304. 33751827 10.1111/febs.15809

[12] M. Scherger , E. Bolli , A. R. P. Antunes , S. Arnouk , J. Stickdorn , A. Van Driessche , H. Schild , S. Grabbe , B. G. De Geest , J. A. Van Ginderachter , L. Nuhn , Cells‐Basel 2020, 9. 10.3390/cells9102222 PMC7600184 33019594

[13] a) B. H. Northrop , S. H. Frayne , U. Choudhary , Polym. Chem. 2015, 6, 3415;

[14] J. Simon , M. Fichter , G. Kuhn , M. Brückner , C. Kappel , J. Schunke , T. Klaus , S. Grabbe , K. Landfester , V. Mailänder , Nano Today 2022, 43, 101375.

[15] M. Brückner , M. Fichter , R. da Costa Marques , K. Landfester , V. Mailänder , Pharmaceutics 2022, 14, 1614. 36015239 10.3390/pharmaceutics14081614 PMC9414227

[16] S. Schmitt , L. Nuhn , M. Barz , H.‐J. Butt , K. Koynov , Macromol. Rapid Commun. 2022, 43, 2100892. 10.1002/marc.202100892 35174569

[17] a) A. Hennig , H. Borcherding , C. Jaeger , S. Hatami , C. Würth , A. Hoffmann , K. Hoffmann , T. Thiele , U. Schedler , U. Resch‐Genger , J. Am. Chem. Soc. 2012, 134, 8268; 22524503 10.1021/ja302649g

[18] a) J. P. Bottcher , C. Reis e Sousa , Trends Cancer 2018, 4, 784; 30352680 10.1016/j.trecan.2018.09.001 PMC6207145

[19] a) R. L. Sabado , S. Balan , N. Bhardwaj , Cell Res. 2017, 27, 74; 28025976 10.1038/cr.2016.157 PMC5223236

[20] a) M. K. Yu , J. Park , S. Jon , Theranostics 2012, 2, 3; 22272217 10.7150/thno.3463 PMC3263514

[21] a) D. V. Haute , J. M. Berlin , Ther Deliv 2017, 8, 763; 28825391 10.4155/tde-2017-0057 PMC6123877

[22] a) S. K. Wculek , F. J. Cueto , A. M. Mujal , I. Melero , M. F. Krummel , D. Sancho , Nat. Rev. Immunol. 2020, 20, 7; 31467405 10.1038/s41577-019-0210-z

[23] J. Simon , G. Kuhn , M. Fichter , S. Gehring , K. Landfester , V. Mailänder , Cells‐Basel 2021, 10. 10.3390/cells10010132 PMC7826990 33445454

[24] S. Tenzer , D. Docter , S. Rosfa , A. Wlodarski , J. Kuharev , A. Rekik , S. K. Knauer , C. Bantz , T. Nawroth , C. Bier , J. Sirirattanapan , W. Mann , L. Treuel , R. Zellner , M. Maskos , H. Schild , R. H. Stauber , ACS Nano 2011, 5, 7155. 21866933 10.1021/nn201950e

[25] E. Papini , R. Tavano , F. Mancin , Frontiers in immunology 2020, 11, 567365. 33154748 10.3389/fimmu.2020.567365 PMC7587406

