# Supplementary material for: Nanobodies Outperform Antibodies – Rapid Functionalization with Equal In Vivo Targeting Properties
Source: Adv Mater. 2024 Oct 29;36(52):2412563. doi: 10.1002/adma.202412563 (PMC11681313; doi:10.1002/adma.202412563)
Supplement: Supplementary file 1 — Supporting Information [file ADMA-36-2412563-s001.docx]

Supporting Information

Nanobodies outperform antibodies – rapid functionalization with equal *in vivo* targeting properties

Carina Jung, Michael Fichter, Jennifer Oberländer, Jenny Schunke, Vanessa Bolduan, Paul Schneider, Jinhong Kang, Kaloian Koynov*, Volker Mailänder*, Katharina Landfester*

Supporting Figures

**Figure S1:** DLS of the unfunctionalized and functionalized samples (as presented in Figure 2).


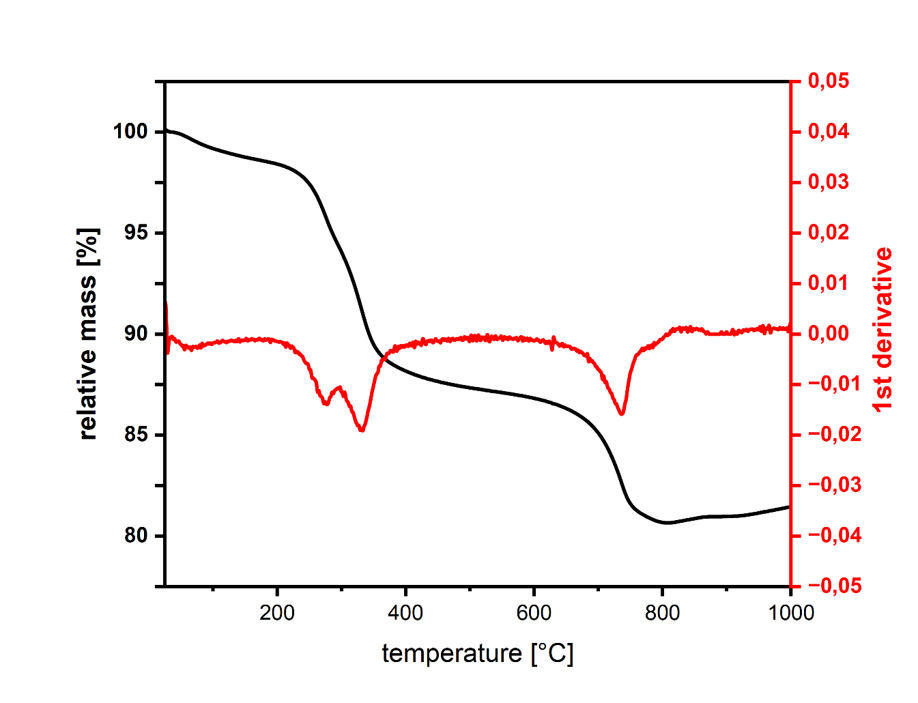


**Figure S2:** TGA results and first derivative of the unmodified mgHES. Results were used to calculate the approximate thickness of the hydroxyethyl starch layer (see calculations).


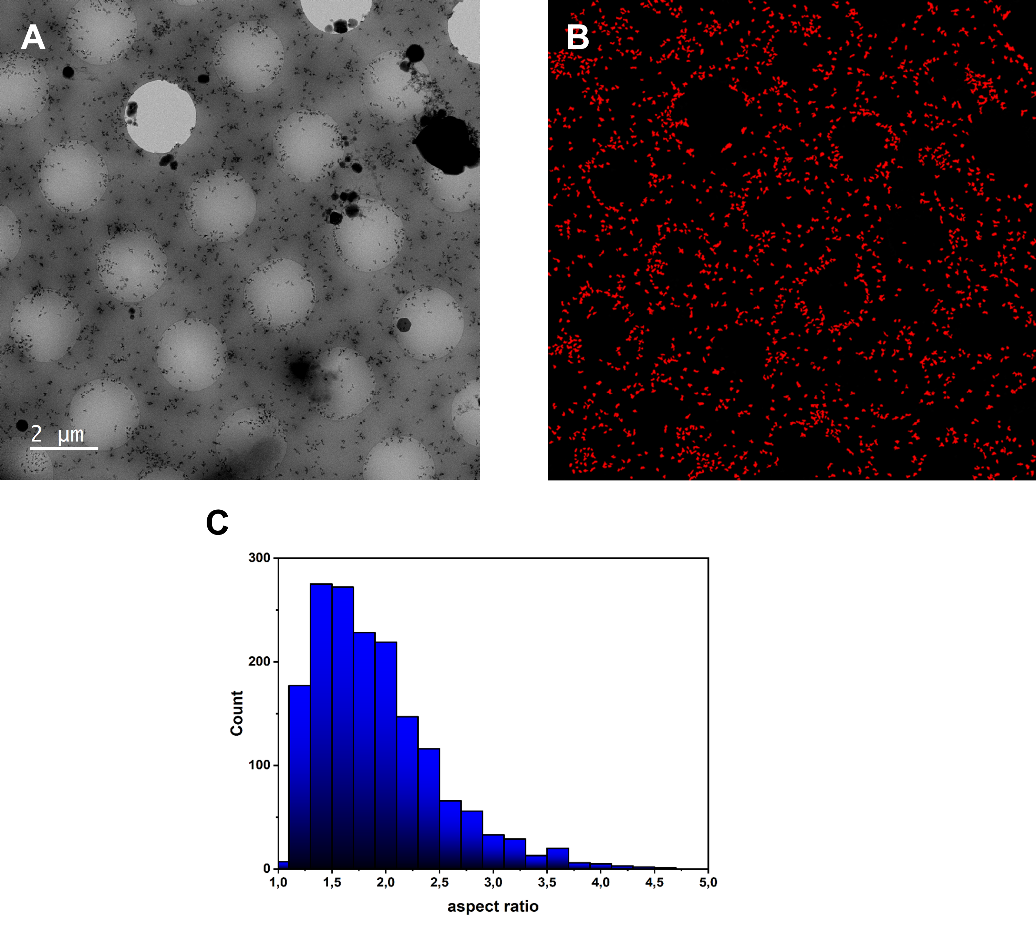


**Figure S3:** Determination of the aspect ratio of mgHES using a cryo-TEM overview image (**A**) and evaluating the nanocarrier dimensions with the software Ilastik (**B**) to find the aspect ratio distribution (**C**).


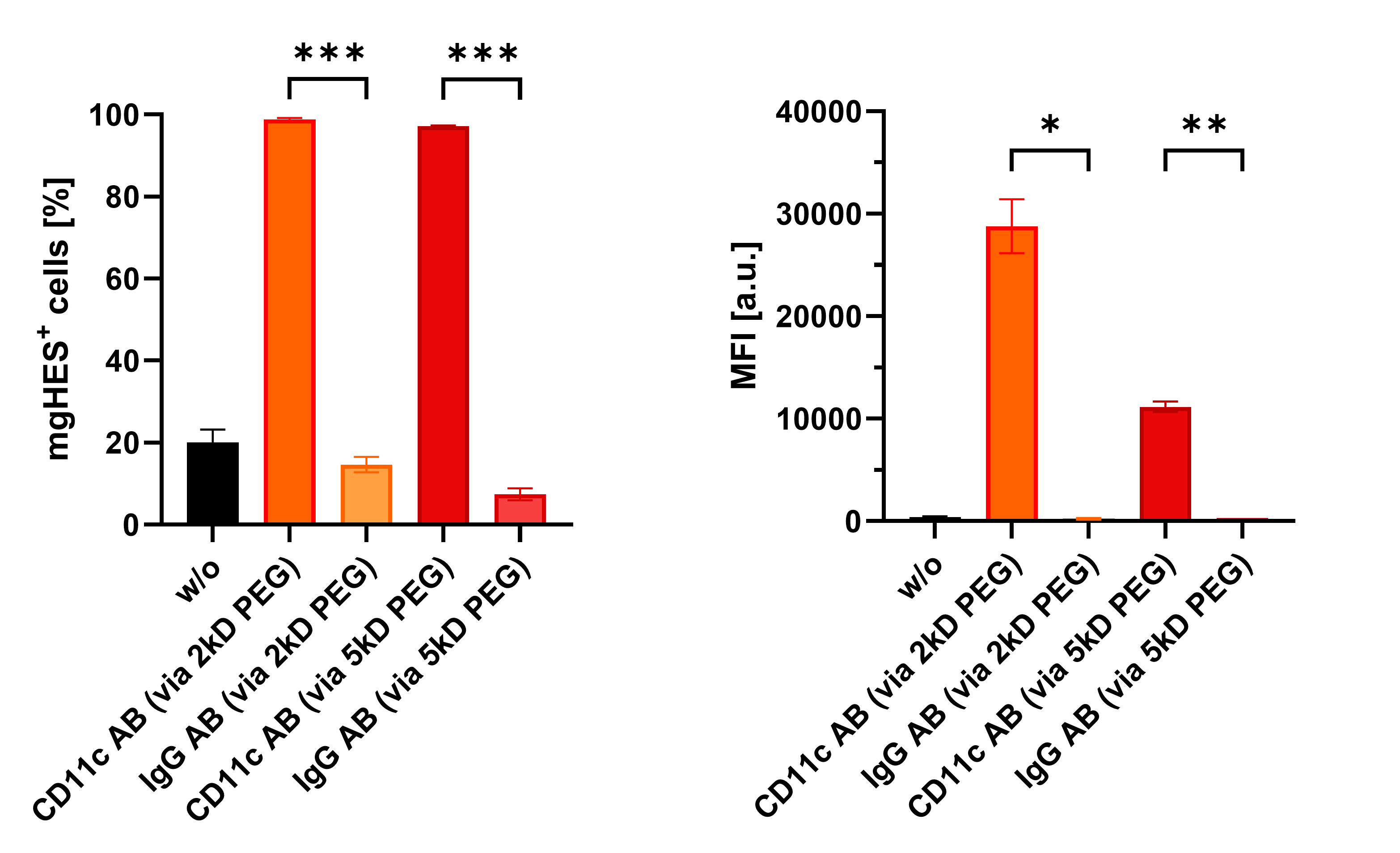


**Figure S4:** Cell uptake results of mgHES-AB with different linker lengths of 2 kDa (PEG_36_) and 5 kDa (PEG_110_). The 2 kDa linker was chosen for further experiments.


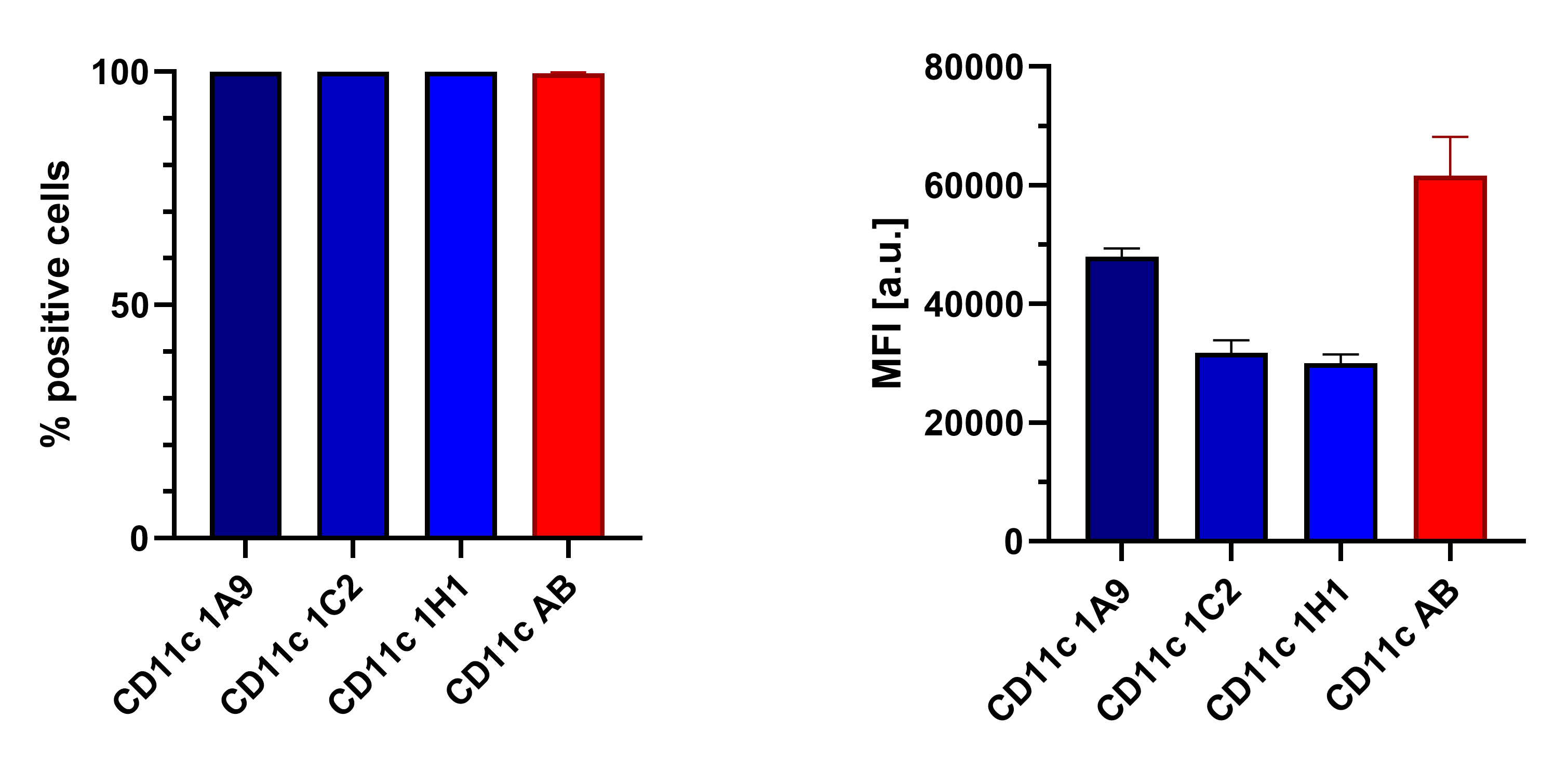


**Figure S5:** Binding test of anti-CD11c nanobody clones 1A9, 1C2, and 1H1, as well as the anti-CD11c antibody on MutuDC1940 cells. The cell samples were incubated with FcR block (CD16/CD32 clone RUO, 5 µg/mL, 15 min, 4 °C), followed by the respective antibody or nanobody at 4 °C for 30 min. After incubation, all samples were washed and incubated with the corresponding secondary antibody (anti-VHH-PE and anti-IgG-DyLight649, 30 min, 4 °C), washed again and analyzed by flow cytometry. As a control, cells were incubated with the secondary antibodies and treated identically.


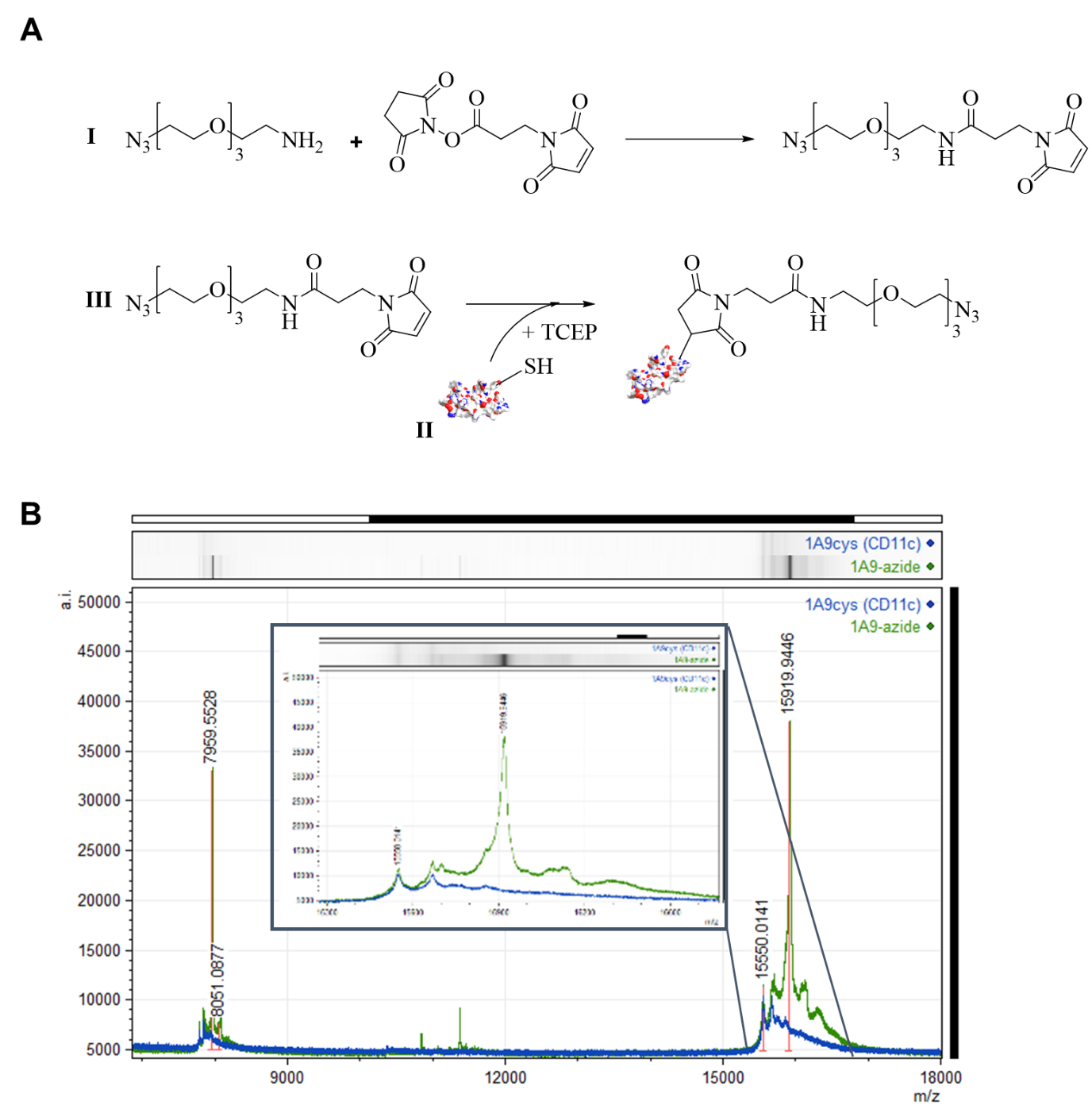


**Figure S6:** mgHES-PEG_n_-NB synthesis and analysis. **A**) azidation of CD11c nanobodies; **B**) MALDI-TOF analysis proves successful linker attachment.


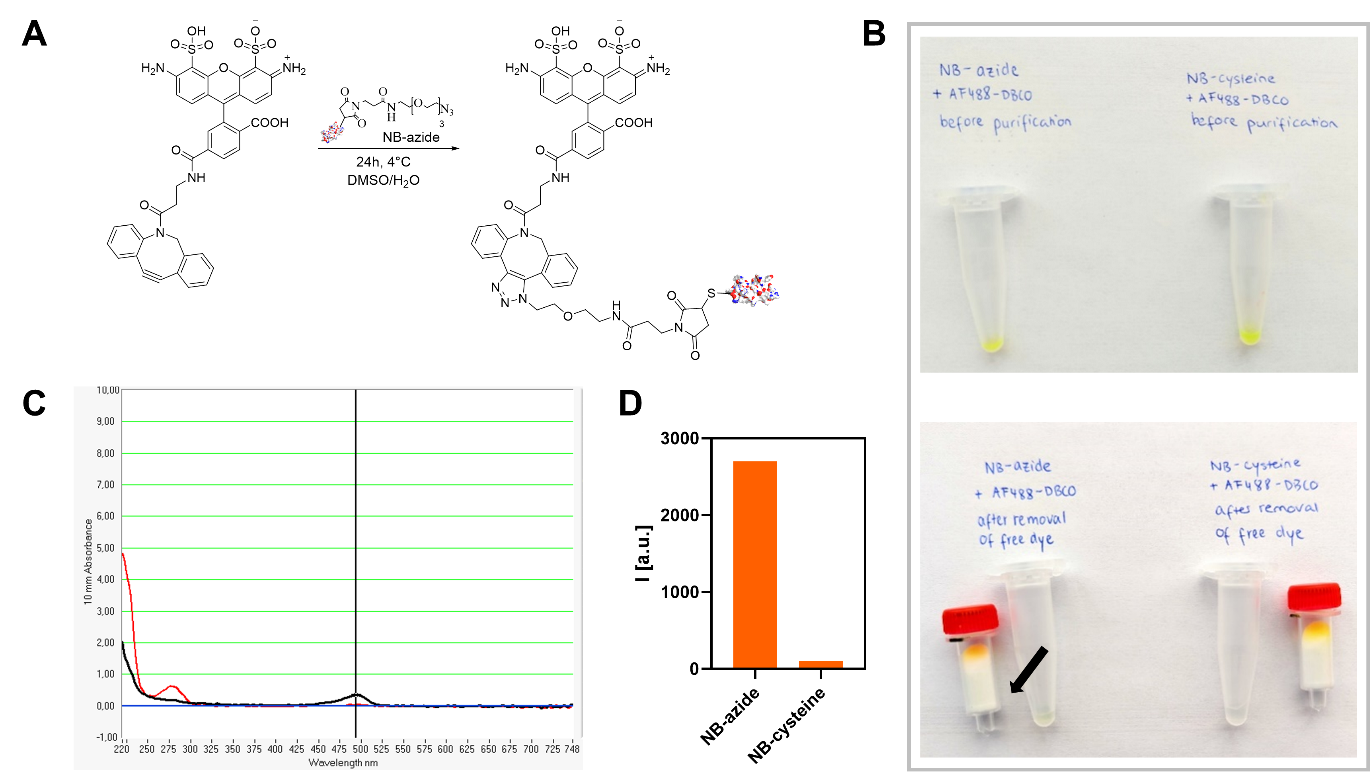


**Figure S7:** mgHES-PEG_n_-NB analysis. **A**) Functionalization of NB-N_3_ with AF488-DBCO; **B**) reaction mixture before (top image) and after (bottom image) purification shows that a yellow tint remains for the sample, but not the control; **C**) fluorescence measurements after purification of NB-AF488 (and a control of unmodified NB treated under similar conditions) show an increased fluorescence that indicates a successful reaction with the dye; **D**) evaluation of the fluorescence of the sample and control.

**Figure S8:** Cell uptake in MutuDC1940 of mgHES-PEG_36_-NB and mgHES-PEG_110_-NB with varying amounts of nanobody, performed in triplicates (30 µg/mL nanocarriers, 2 h incubation period).


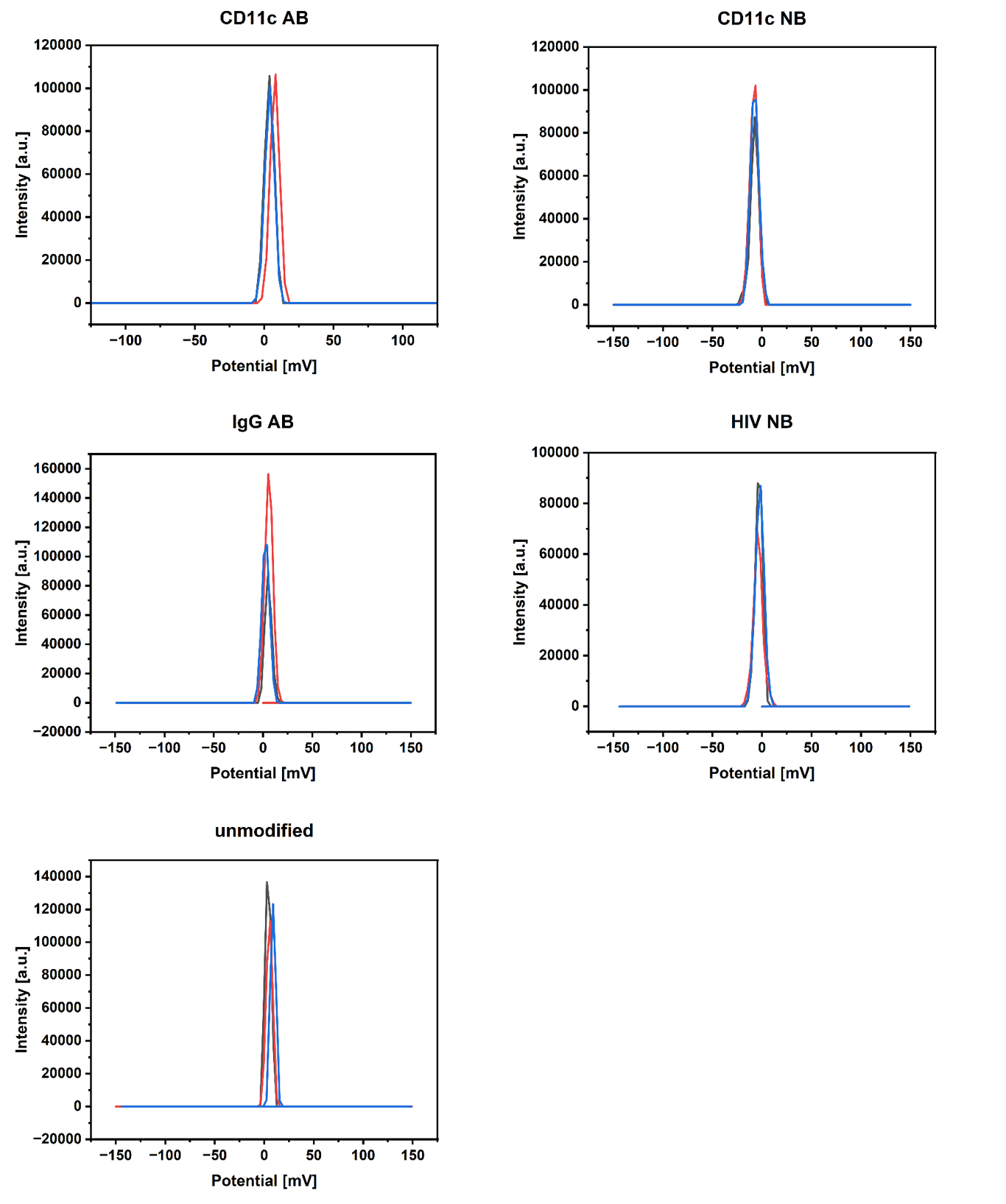


**Figure S9:** Zeta potential results for the unmodified and modified samples (see Figure 2).


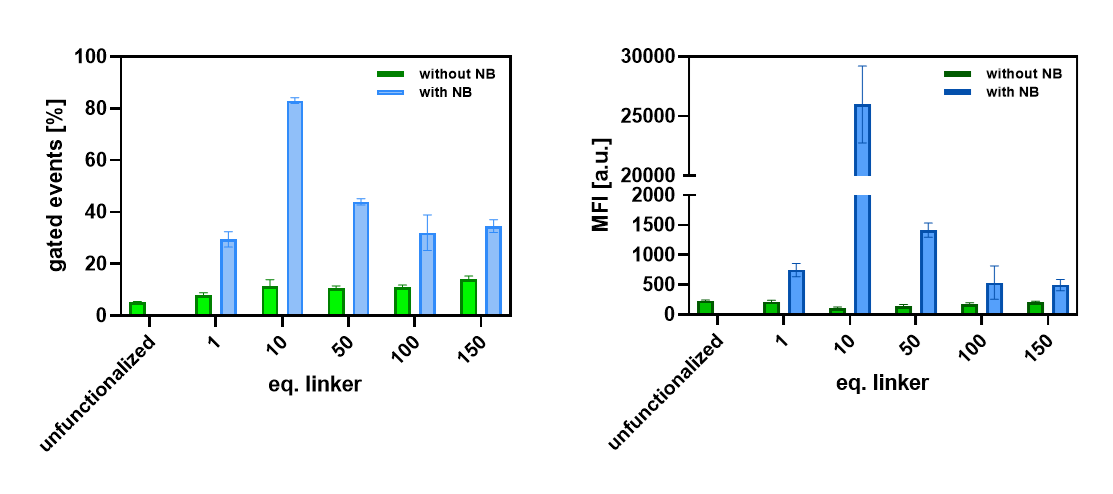


**Figure S10:** Investigation of the attached nanobody amount on samples functionalized with identical nanobody amounts, but varying linker amounts *via* secondary antibody and nanocarrier-only flow cytometry.


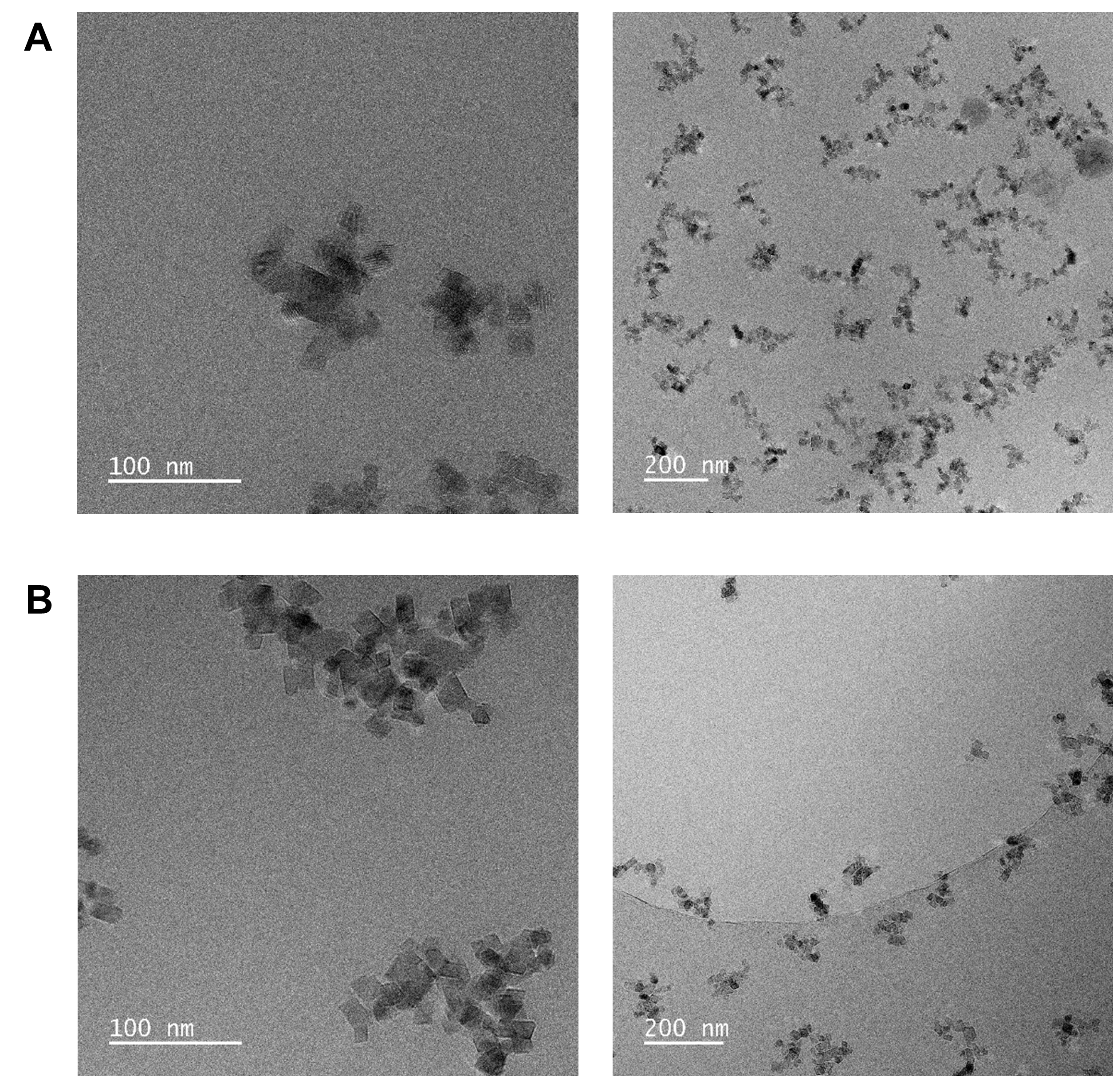


**Figure S11:** Cryo-EM images of mgHES before (**A**) and after (**B**) functionalization with CD11c nanobodies.

**Figure S12:** Additional data to Figure 3: nanoparticle flow cytometry with an AF647-secondary antibody targeting nanobodies.


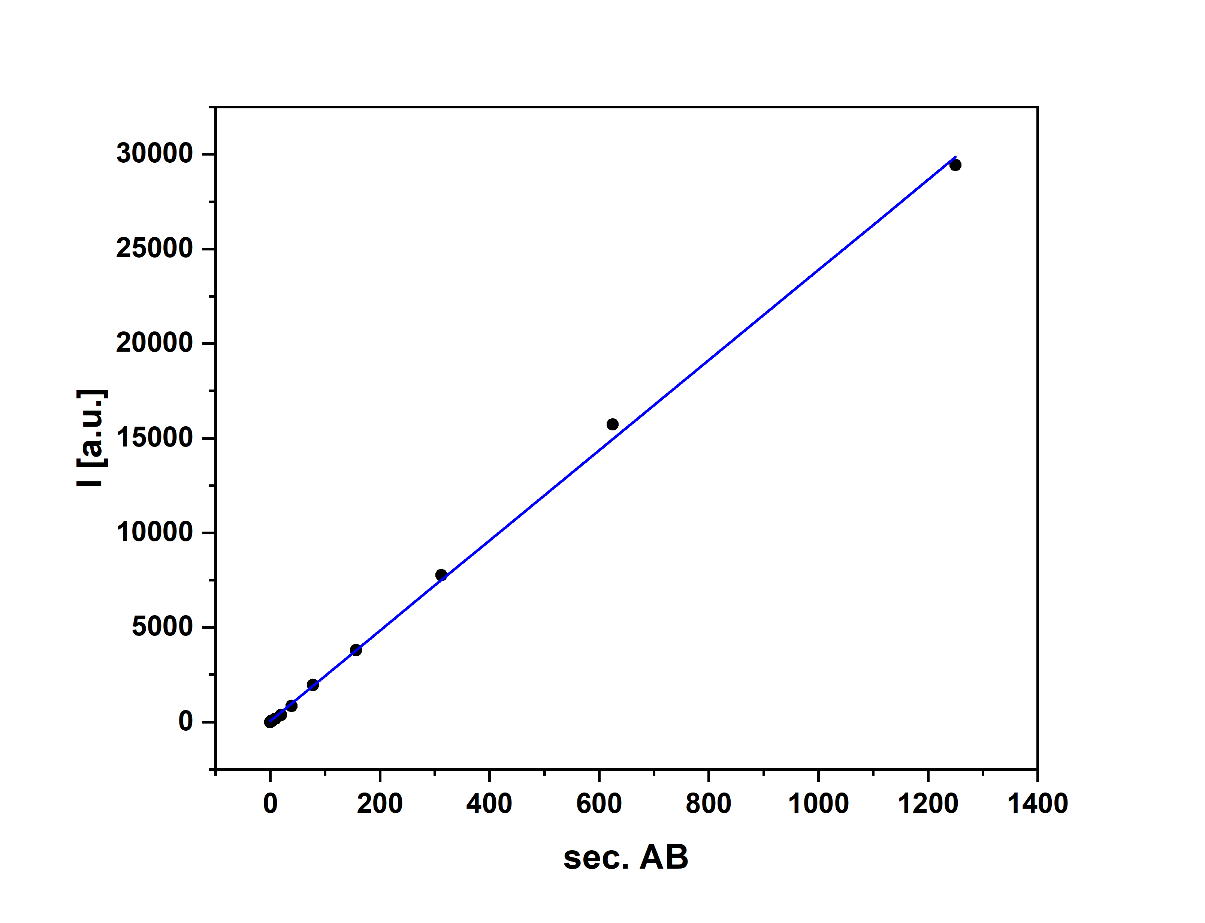


**Figure S13:** Calibration curve for Figure 3D. Standards of defined concentrations of AF647-tagged secondary antibody were measured for this fluorescence calibration. The resulting calibration curve was used to calculate the concentration in the samples.


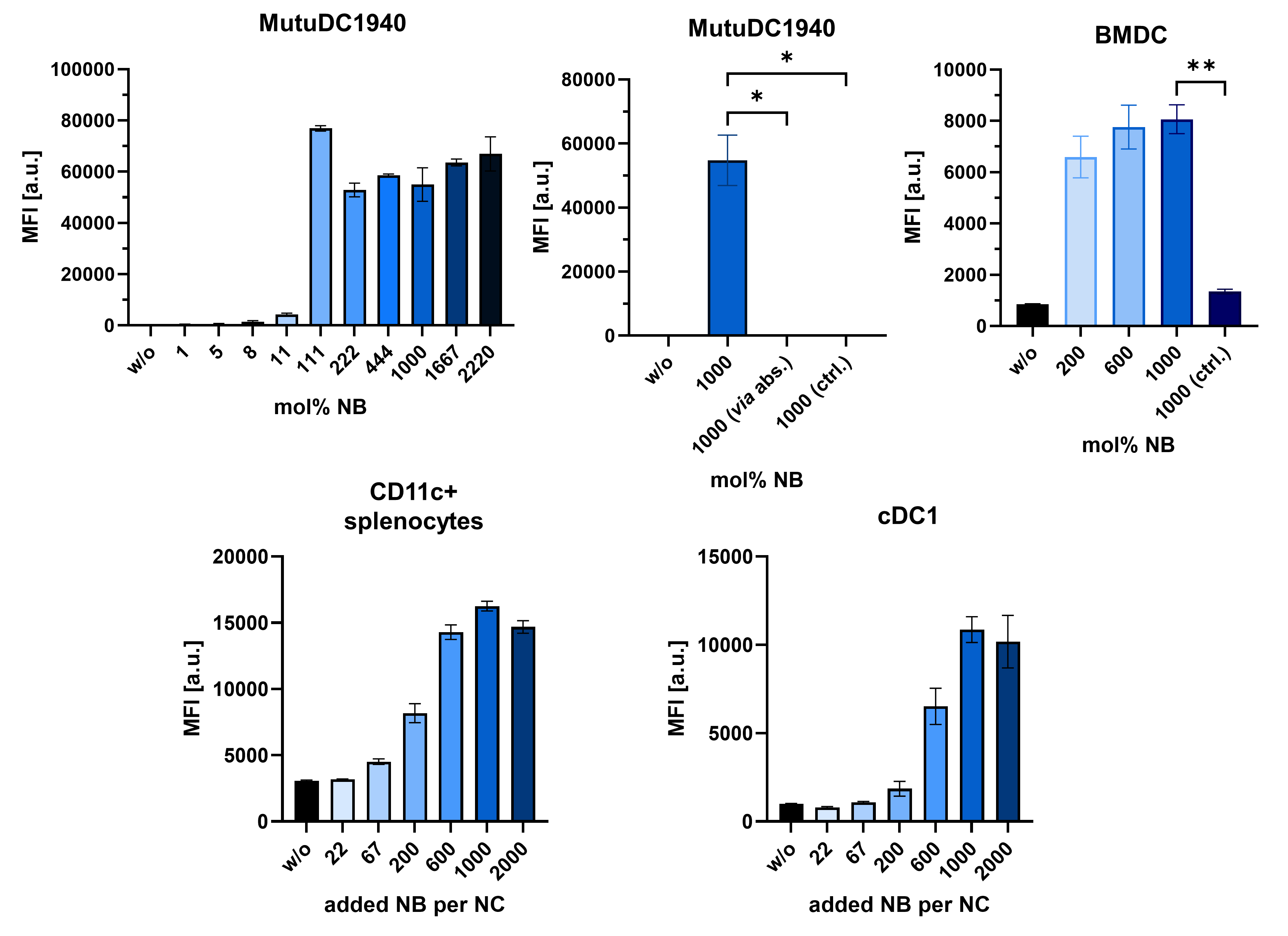


**Figure S14:** Corresponding MFIs to Figure 4.

**Figure S15:** Additional data to Figure 4: cDC2 uptake.


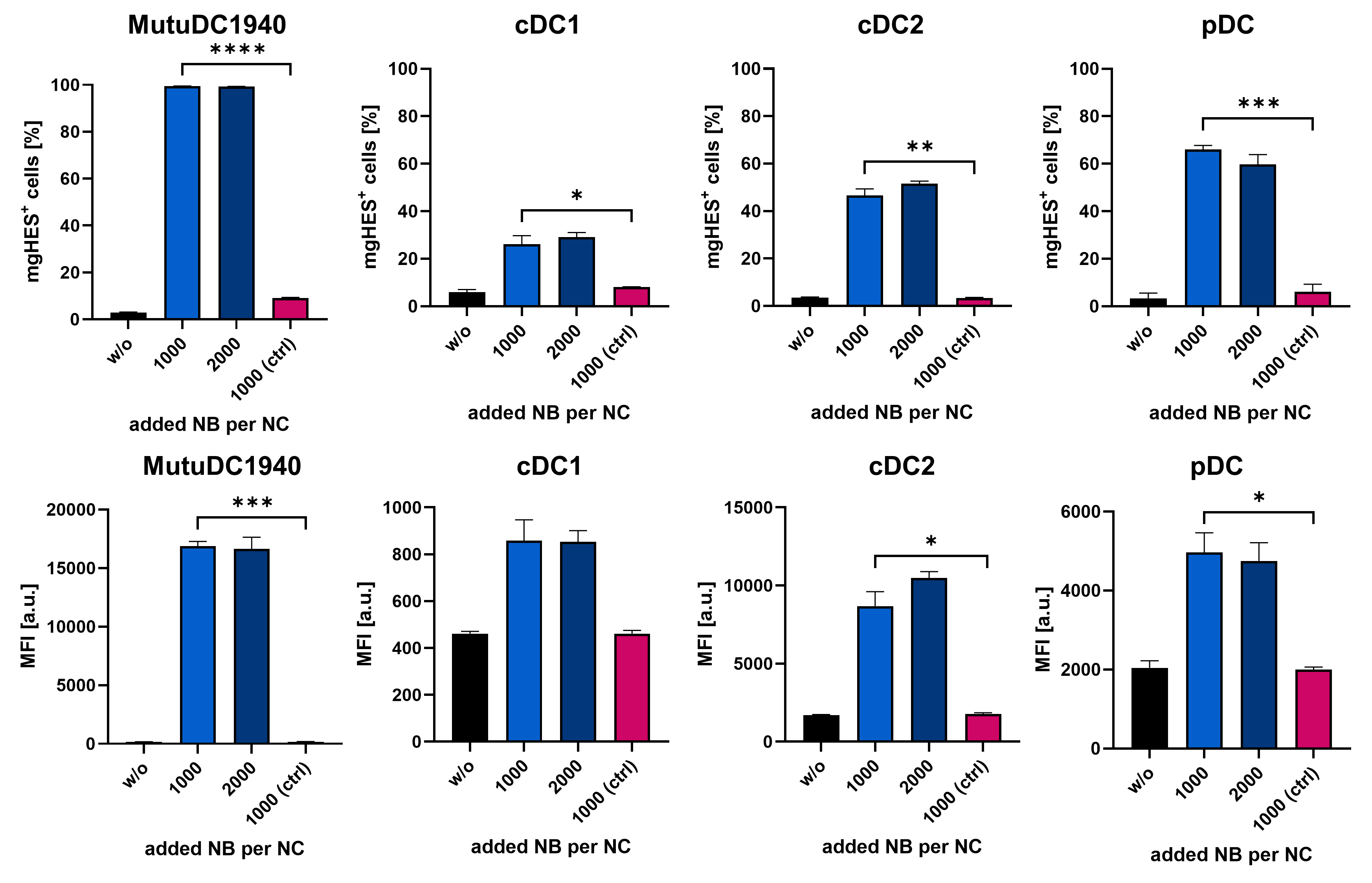


**Figure S16:** Cell uptake results of samples functionalized with 1000 and 2000 NB/NC, as well as 1000 NB/NC without prior linker attachment as an absorption control. Conditions identical to Figure 4.


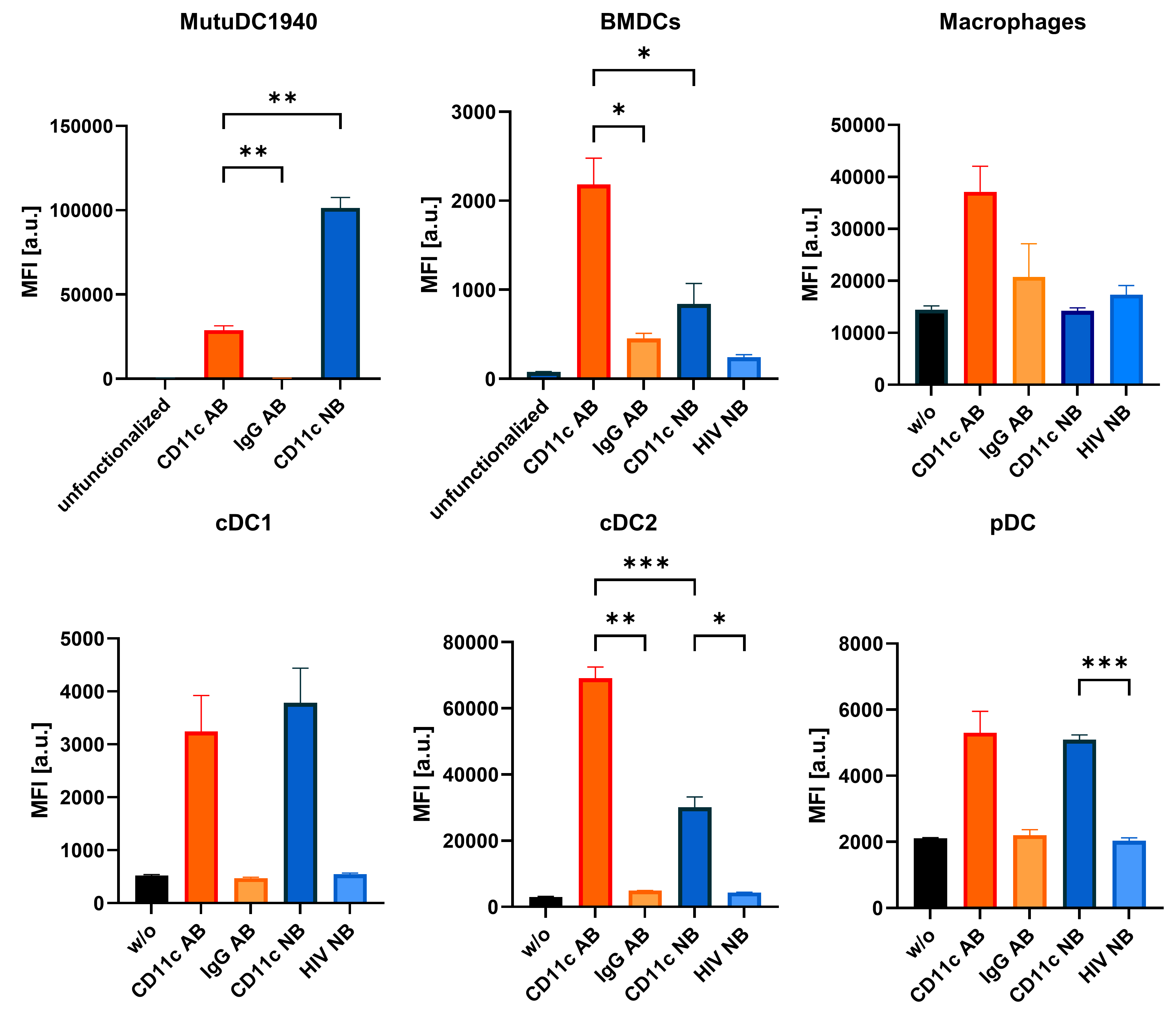


**Figure S17:** Corresponding MFIs to Figure 5.


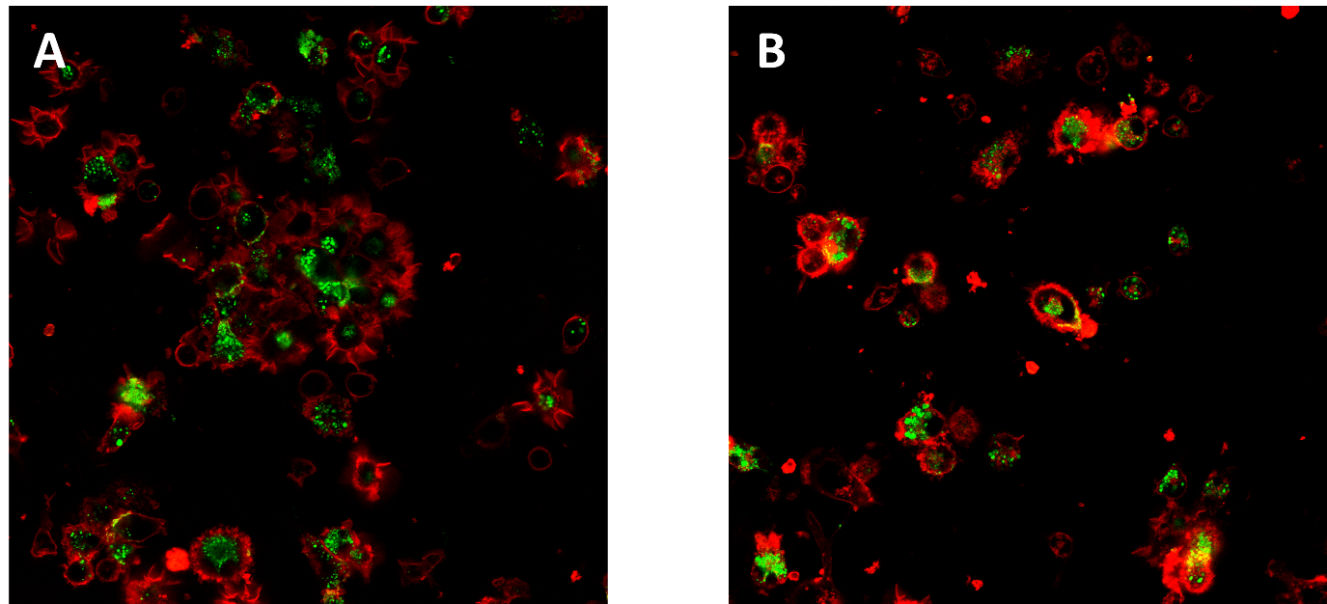


**Figure S18:** cLSM evaluation of the uptake of CD11c-targeting nanocarrier samples into BMDCs. Cell uptake was performed at 30 µg/mL for 24 h. Staining of the cell membrane was performed using CellMask^TM^ DeepRed. **A)** mgHES-CD11c NB; **B)** mgHES-CD11c-AB.


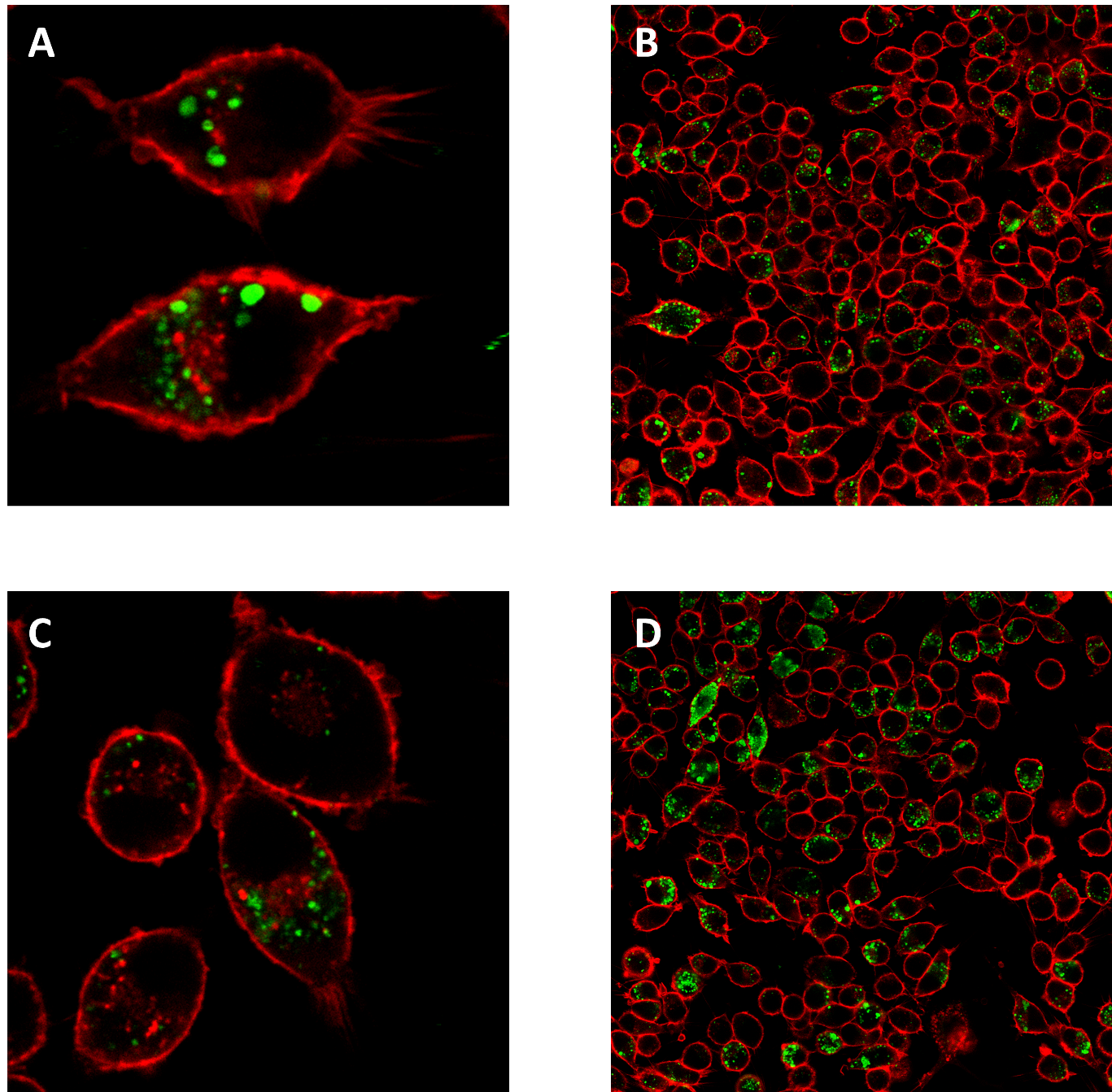


**Figure S19:** cLSM evaluation of the uptake of CD11c-targeting nanocarrier samples into DC2.4. Cell uptake was performed at 30 µg/mL for 24 h. Staining of the cell membrane was performed using CellMask^TM^ DeepRed. **A-B)** mgHES-CD11c NB; **C‑D)** mgHES‑CD11c‑AB.


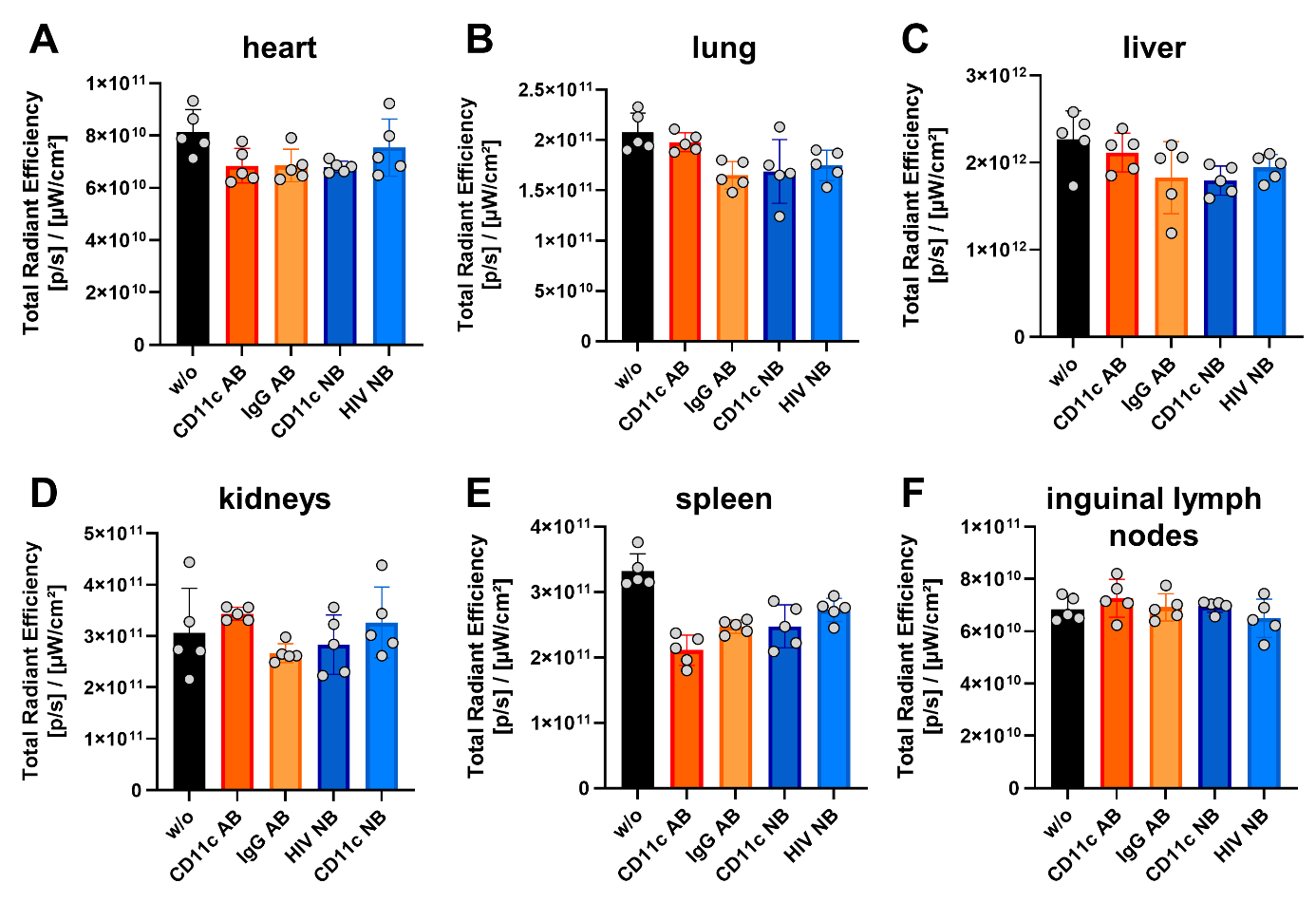


**Figure S20:** Biodistribution of mgHES nanocarrier formulations into different organs. **A) ‑ F)**Animals were intravenously injected with 500 µg of mgHES nanocarriers. Heart (**A**), lung (**B**), liver (**C**), kidneys (**D**), spleen (**E**), and inguinal lymph nodes (**F**) were dissected 24 h post injection and imaged using the IVIS® SpectrumCT. The fluorescence intensity of all organs was analyzed.


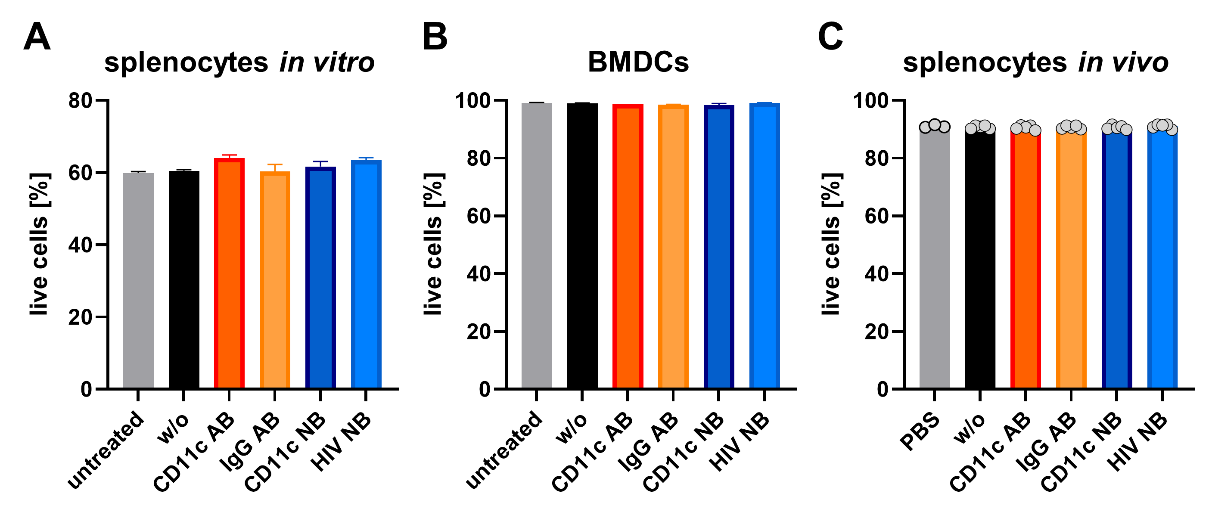


**Figure S21**: Flow cytometric determination of live cells after incubation with mgHES nanocarriers or after intravenous injection. Splenocytes (**A**) or BMDCs (**B**) were harvested 24 h after incubation with 30 µg/mL mgHES nanocarrier formulations or splenocytes were isolated from mice 24 h after injection with 500 µg of nanocarriers (**C**). Dead cells were discriminated from living cells by adding 100 µL Live/Dead Aqua diluted 1:1000 in PBS for 20 min at 4 °C.


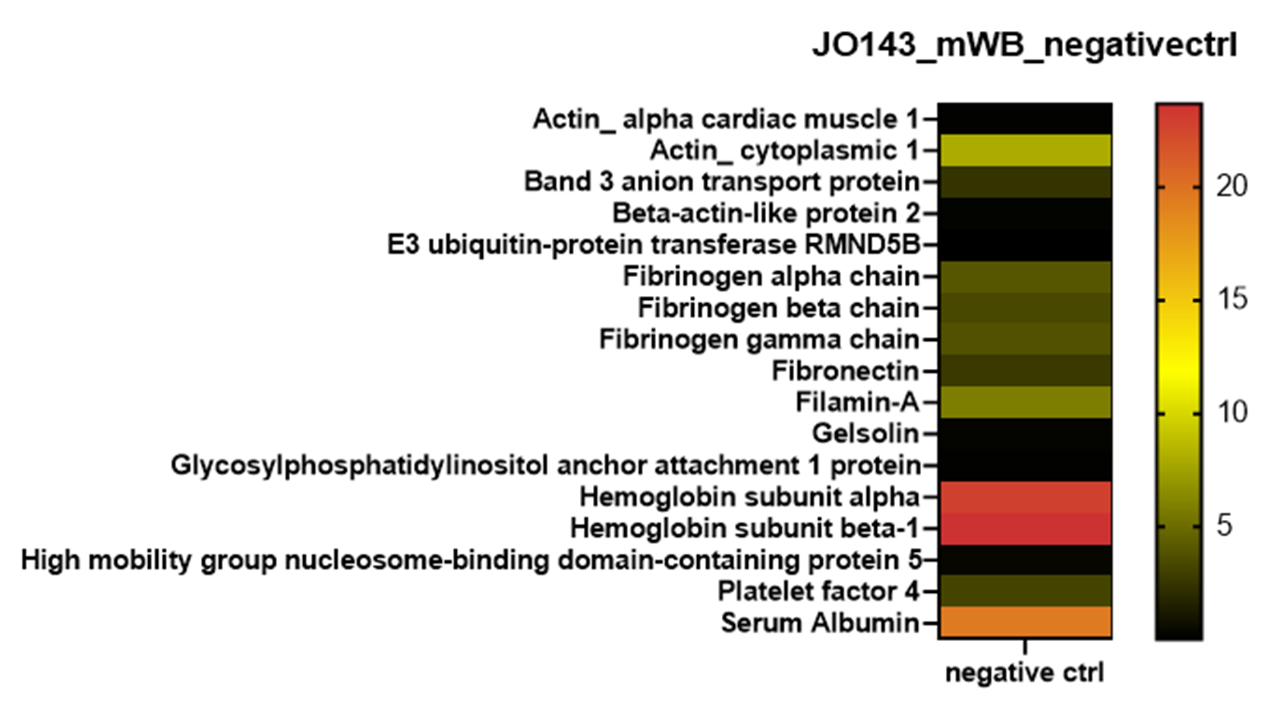


**Figure S22:** Murine full blood protein corona control.

Supporting Calculations

**Table S1:** Cost evaluation of the antibody and nanobody functionalization strategies. Prices were calculated per nmol amine groups, disregarding the cost for the unmodified nanocarrier (system is applicable for all types of nanocarriers with amine surface groups) and protein (system can be adapted for a large variety of antibodies/nanobodies with varying cost).

| **reagent** | **Cost per mass (supplier)*** | **Required amount for functionalization per nmol NH_2_** | **Cost for the functionalization per nmol NH_2_** | **Total cost for functionalization per nmol NH_2_** | **Total synthesis time** |
| --- | --- | --- | --- | --- | --- |
| **TCEP** | 142€ per 2g (Merck) | 0.5 µg | 0.00003€ | **0.03€** | **Ca. 5 hours** |
| **Sulfo-SMCC** | 355€ per 50 mg (ThermoFisher) | 4.36 µg | 0.0284€ |  |  |
| **Site-Click Kit**** | 496€ per Kit (ThermoFisher) | 1/30 Kit | 16.53€ | **17.97€** | **Ca. 5 days** |
| **NHS-PEG_2k_-DBCO** | $385 per 25 mg (Nanocs) | 0.1 mg | $1.54 = 1.44€ |  |  |

*all prices were searched for on June 14^th^, 2024 **yield per kit is about 150 µg AB-N_3_

**Thickness of the HES layer around the magnetite core**

According to TGA: 87 wt% magnetite, 13 wt% HES

r = r_1_ + r_2_ = 85 nm (according to DLS, assuming spherical NCs)

V = 4/3 π r³ = V_1_ +V_2_

ρ_1_ = ρ_Fe3O4_ = 5.2 g/cm³, ρ_2_ = ρ_HES_ = 1.5 g/cm³

For 100g NCs:

m_1_ + m_2_ = 87g Fe_3_O_4_ + 13g HES

V_1_ + V_2_ = 17cm³ Fe_3_O_4_ + 8.7 cm³ HES

V_1_/V_2_ = 8.7/17 ≈ 0.512 V_2_ = 0.512 V_1_ V = 1.512 V_1_

4/3 π r³ = 1.512 x 4/3 π r_1_³

r = 1.512^1/3^r_1_

r_1_ = r x 1.512^-1/3^ ≈ 74 nm

r_2_ = r – r_1_ ≈ 11 nm

**Calculation of surface area per nanobody for spherical (A) nanocarriers and under consideration of the aspect ratio (B)**

d_mgHES_ = 170 nm, r = 85 nm

1. Assume sphere: A = 4πr² = 90 792 nm², with 90 NB per mgHES: ca. 1008nm²/NB
2.
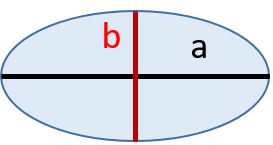
Include aspect ratio of the mgHES: assume an oval with a/b = 3/2 (a = 1.5b), (a+b)/2 = 170 nm 🡪 b = 156 nm, a = 204 nm. A = 92 546 nm² (calculated with https://rechneronline.de/pi/ellipsoid.php), with 90 NB per mgHES: ca. 1028 nm²/NB

Materials

*mgHES.* mgHES (BNF-Starch-RedF) were purchased from Micromod and characterized by DLS, Zeta potential, TEM and TGA. They consist of a magnetic iron oxide core, surrounded by a hydroxyethyl starch shell, and are functionalized with a fluorescent dye (Dy555, Dyomics). ex. 552 nm / em. 580 nm.

*Antibody azidation.* Antibodies were modified using a *Site Click^TM^ Antibody Azido Modification Kit* (Thermo Fisher Scientific). In accordance with the instructions provided, the galactose residue of the carbohydrate domain at the Fc region was cleaved *via* β-galactosidase (overnight, 37 °C). In a second step, azide groups were attached *via* a combination of the GalT (Y289L) enzyme and a UDP-GalNAz donor (overnight, 30°C). Purification and concentration was achieved by centrifugation and washing with amicon centrifugal filters. The final concentration was determined in a Pierce 660 nm assay.

*mgHES-AB.* In a 1.5 mL reaction vessel, 50 eq. NHS-PEG_36_-DBCO (2 kDa) were added to mgHES (10 mg/mL in PBS, 3 nmol/mg NH_2_) and the reaction mixture was shaken at room temperature overnight to form mgHES-DBCO. Purification was achieved *via* washing with PBS and magnetic separation (3x). The concentration was determined by fluorescence calibration (ex. 552 nm / em. 580 nm). 1.5 wt% (3.3 mol% AB or 13 mol% azide groups when assuming 100% functionalization in the first step) AB-N_3_ were added, and the solution was shaken at room temperature overnight (500 rpm). The antibody-modified samples were purified *via* washing with PBS and magnetic separation. The final concentration was determined *via* fluorescence calibration (ex. 552 nm / em. 580 nm).

*mgHES-NB.* In a 1.5 mL reaction vessel, 10 eq. sulfo-SMCC were added to mgHES, and the reaction mixture was shaken at room temperature and 500 rpm for one hour to form mgHES‑maleimide. Purification was achieved *via* washing with PBS and magnetic separation (3x), and the concentration was determined by fluorescence calibration (ex. 552 nm / em. 580 nm).

Simultaneously, nanobodies were treated with 10 wt% (6 eq.) TCEP (rt, 500 rpm, 1 h) and then purified using Zeba spin desalting columns (7 kDa MWCO). The concentration was determined using a *Nanodrop8000* device.

1.5 wt% (33 mol% when assuming a functionalization degree of 100% due to the large linker excess) of the treated nanobody carrying now exposed thiol groups in form of a C-terminal cysteine tag were added. The samples were shaken at 500 rpm and room temperature for three hours. Purification was achieved by washing with PBS and magnetic separation (3x), and the concentration was determined *via* fluorescence calibration (ex. 552 nm / em. 580 nm).

*NB azidation.* Nanobodies were treated with 10 wt% (6 eq.) TCEP (rt, 500 rpm, 1h) and then purified using Zeba spin desalting columns (7 kDa MWCO). The concentration was determined using a *Nanodrop8000* device.

Simultaneously, N_3_-PEG_3_-maleimide was synthesized *in situ* as follows. 1.1 eq. of N_3_-PEG_3_-NH_2_ were dissolved in 1 mL dry DMF under inert atmosphere and shaken for 30s. NHS-mal (1 eq.) was added dropwise under inert atmosphere, and the reaction mixture was shaken at room temperature for 30 min. The final solution of N_3_-PEG_3_-maleimide had a concentration of 75 nmol/µL and was used without further purification.

20 eq. N_3_-PEG_3_-maleimide were added to the CD11c nanobody clone and the mixture was shaken at 500 rpm and room temperature for four hours. Excess linker was removed *via* Zeba spin desalting column (7 kDa MWCO), and the concentration was determined using a *Nanodrop8000* device.

*NB-AF488.* AF488-DBCO was dissolved in dry DMSO to form a 5 mg/mL (6.3 mM) solution. 0.75 µL (3.75 µg, 4.73 nmol, 1.6 eq.) of this solution were added to 45 µg (3 nmol) nanobody and the reaction was kept at 4 °C overnight in the dark. Purification was achieved *via* Zeba Spin Desalting Column (7 kDa MWCO) and the product was investigated using nanodrop and platereader.

*mgHES-NB via NB-azide.* In a 1.5 mL reaction vessel, 50 eq. NHS-PEG_36_-DBCO (2 kDa) were added to mgHES (10 mg/mL in PBS, 3 nmol/mg NH_2_) and the reaction mixture was shaken at room temperature overnight to form mgHES-DBCO. Purification was achieved *via* washing with PBS and magnetic separation (3x). The concentration was determined by fluorescence calibration (ex. 552 nm / em. 580 nm). Various amounts of NB-N_3_ (3333, 1667, 1000, and 500 NB/NC) were added, and the solution was shaken at room temperature overnight. The nanobody-modified samples were purified *via* washing with PBS and magnetic separation. The final concentration was determined *via* fluorescence calibration.

*Validation of attachment via secondary antibody.* To 17 µL and 2 µL of 1 µg/µL sample in PBS, 1 µL AF647-goat-α-VHH (1.7 µg/µL) were added, and the samples were incubated in the dark at 4 °C for 30 min. 1000 µL PBS were added, and the samples were evaluated using flow cytometry.

*Quantification of the attached nanobody amount by FCS.* To a nanocarrier sample prepared with 1000 nanobodies per nanocarrier, a Fab fragment mixture with 2.2% dye-labelled Fab fragments in unlabeled Fab fragments was added in excess. The mixture was incubated (4 °C, overnight, in the dark). Unbound Fab was removed by washing (PBS) and magnetic separation. Using FCS, two bound dye-tagged Fab fragments per nanocarrier were detected. Since only 2.2% of Fab fragments are fluorescent, this means that a total amount of 2/0.022 = 90 nanobodies per nanocarrier was determined.

*Quantification of the attached nanobody amount (inverse experiment).* To 50 µg samples functionalized with an initial amount of 600, 1000, and 2000 nanobodies per nanocarrier, 1 eq. of AF647 functionalized secondary antibody was added. The volumes were adjusted to 100 µL, and the samples were incubated overnight (4 °C, in the dark). All samples were centrifuged (5000 g, 15 min, rt). The supernatants were removed and centrifuged (5000 g, 15 min, rt) again to make sure no more nanocarriers were present. Standard solutions of the secondary antibody were prepared, and the prepared and used to determine the concentration of secondary antibody in the supernatants by fluorescence calibration. The amount of nanobodies bound on the nanocarrier surface was calculated as the difference between the initially added amount of secondary antibody and the amount measured in the supernatants.

*Ex vivo protein corona.* Blood was isolated form C57BL/6 mice *via* cardiac puncture and supplemented with heparin. 1 mg nanocarriers were incubated in 2 mL blood for 1 min and afterwards recovered by magnetic separation. Purification was achieved *via* washing with PBS (3x) and magnetic separation. Strongly attached proteins remained on the nanocarrier surface and were desorbed using 2 wt% SDS in an aqueous 62.5 mM TrisCl solution and heating to 95 °C for 5 min, followed by magnetic separation. The remaining supernatant containing the hard protein corona was analyzed *via* LC-MS.

*In solution digestion.* SDS was removed from the samples using Pierce Detergent Removal Spin Columns (Thermo Fisher Scientific). The remaining proteins were precipitated using a ProteoExtract protein precipitation kit (CalBioChem), isolated by centrifugation (14 000 g, 10 min) and suspended with 50 mM RapiGest SF (Waters) in ammonium bicarbonate buffer. 5 mM dithiothreitol was added to reduce the proteins (45 min, 56 °C), followed by an alkylation with 15 mM iodoacetamiede (1 h, rt). The proteins were digested using 0.02 eq. trypsin at 37 °C for a duration of 18 h. The reaction was quenched with HCl_aq_. Purification was achieved *via* centrifugation (14 000 g, 15 min, 4 °C), and the samples were evaluated using LC‑MS.

Methods

*DLS.* Particle sizes were measured on a *Zetasizer Nano-S90* device (Malvern, Germany) at 20 °C.

*Zeta potential.* Zeta potentials were determined using a *Zetasizer Nano Z* device (Malvern, Germany) at 20 °C.

*Cryo-TEM.* 3 μl of sample was pipetted onto a quantifoil 2/1 300 mesh copper grids (previously glow discharged). The grid was plunged into liquid ethane with a Vitrobot IV and transferred in liquid nitrogen to a Titan Kirios G4 Cryo-TEM. The TEM was operated at 300 kV with 70μm objective aperture. Micrographs were acquired using a Gatan K3 detector with a 20eV filter.

*Determination of the Aspect Ratio.* A total of one micrograph (pixel size of 4 nm, image size of 3456 x 3456 pixels) was analyzed using the image analysis software Ilastik (Lit). Approximately 1700 individual particles were positively identified and their aspect ratio was determined from the maximum and minimum elliptical diameters.

*Flow Cytometry.* Flow cytometric experiments were performed on an *Attune^TM^ NxT* flow cytometer (Thermo Fisher Scientific). The signals were recorded using the YL1 channel for Dy555 (excitation at 561 nm, band pass filter 585/16 nm), and the RL1 channel for AF647 (excitation at 638 nm, emission filter 670/14 nm), respectively. Cell debris was excluded in the Attune^TM^ NxT software by selection of a cell population in the FSC/SSC scatter plot. The percentage of dye-positive cells and the corresponding median fluorescent intensity (MFI) were considered for the evaluation.

*Fluorescence Correlation Spectroscopy.* Fluorescence correlation spectroscopy experiments were performed on a LSM 880 device (Carl Zeiss, Jena, Germany). The excitation was performed with the 633 nm line of a HeNe laser focused into the studied solutions through a C-Apochromat 40×/1.2 W water immersion objective (Carl Zeiss, Jena, Germany). The emission light was collected with the same objective and, after passing through a confocal pinhole, directed to a spectral detection unit (Quasar, Carl Zeiss), in which a detection range 640 – 700 nm was selected. Eight-well polystyrene chambered cover glasses (Nunc™ Lab-Tek™, Thermo Fisher Scientific, Waltham, MA, USA) were used as sample cells for the studied solutions. Series of 20 FCS measurements with a total duration 200 s were performed. The time-dependent fluctuations of the fluorescent intensity *δI(τ)* were recorded and analyzed by an autocorrelation function *G(τ)* = 1 *+* *δI(τ)* · *δI(τ + τ) >/<I(τ)>*^2^. The obtained in this way experimental autocorrelation curves were fitted with the analytical expression the autocorrelation function of an ensemble of freely diffusing fluorescence species:

$G(\tau)=1+\frac{1}{N}\left[ 1+\frac{f_{T}}{1-f_{T}}e^{-\tau/\tau_{T}} \right]\frac{1}{\left[ 1+\frac{\tau}{\tau_{D}} \right]\sqrt{1+\frac{\tau}{S^{2}\tau_{D}}}}$ (eq.S1)

Here, *N* is the average number of diffusing fluorescence species in the observation volume, *f_T_* and *τ_T_* are the fraction and the decay time of the triplet state, *τ_D_* is the diffusion time of the species and *S* is the so-called structure parameter, *S* = *z*_0_/*r*_0_, where *z*_0_ and *r*_0_ represent the axial and radial dimensions of the confocal volume, respectively. Furthermore, the diffusion time, *τ_D_*, is related to the respective diffusion coefficient, *D*, through: $\tau_{D}=\frac{r_{0}^{2}}{4D}$*.* The fits yielded the corresponding diffusion times, and subsequently the diffusion coefficients and through the Stocks-Einstein relation the hydrodynamic radius of the fluorescent species. Furthermore, the fluorescent brightness of the species FB=*<I(τ)>/N*A was also obtained from the fits. As the value of *r*_0_ depends on the specific characteristics of the optical setup, a calibration experiments were performed using a fluorescent tracer with known diffusion coefficient, i.e., Alexa Fluor 633 in water.

*Concentration via Fluorescence Calibration.* Nanocarrier concentrations were determined by fluorescence concentration. A calibration curve was made using mgHES in concentrations ranging from 1.000‑0.03125 mg/mL in PBS. Samples were measured in duplicates and in 1:100 and 1:200 dilution. The fluorescence intensities were measured using a *Tecan Infinite M1000* plate reader (ex. 552 nm / em. 580 nm).

*Concentration via Nanodrop.* Nanobody concentrations were measured using a *Nanodrop8000* device in accordance with the instructions provided in the manual.

*Concentration via Pierce Assay.* Protein quantification was performed by the Pierce 660 nm assay (Thermo Fisher, Germany), following the instructions provided by the supplier. Bovine serum albumin was used as a standard (calibration curve with concentrations of 2.000‑0.03125 mg/mL in PBS). Absorption was measured with a *Tecan Infinite M1000* plate reader.

*Liquid Chromatography Mass Spectrometry (LC-MS).* LC-MS measurements were performed using a *nanoACQUITY UPLC* system coupled to a *Synapt G2-*Si mass spectrometer, as previously described.

*Cell Culture of MutuDC1940s.* MutuDC1940 cells were cultured in Iscove's Modified Dulbecco's Medium (IMDM), supplemented with 10% heat-inactivated FBS, 5 mM HEPES, 1% P/S, 55 µM ß-mercaptoethanol amd 1% GlutaMax. The cells were kept at 37 °C and 5% CO_2_ in a humidified incubator, using 5 mM EDTA / 10 mM HEPES for passaging and harvesting. Viability and cell cound were determined from a 1:1 dilution with trypan blue using an automated cell counter (TC10, Bio-Rad, Germany). The passage was kept below 20 to ensure stability.

*Cell Culture of DC2.4s.* DC2.4 cells were cultured in IMDM, supplemented with 5% FBS, 1% P/S, 55 µM ß-mercaptoethanol amd 1% GlutaMax. The cells were kept at 37 °C and 5% CO_2_ in a humidified incubator, using 2 mM EDTA in PBS for passaging and harvesting. Viability and cell cound were determined from a 1:1 dilution with trypan blue using an automated cell counter (TC10, Bio-Rad, Germany). The passage was kept below 20 to ensure stability.

*Animals.* 6-12 week-old C57BL/6J mice were obtained from Charles River Laboratories (Germany) and kept until experimental procedures at the University Medical Center Mainz (Germany) with food and water supply ad libitum according to the “Guide for Care and Use of Laboratory Animals”. The performed experiments were approved by the local animal welfare authority (“Landesuntersuchungsamt Rheinland-Pfalz”) and *in vivo* studies were conducted under the approval number G 20-1-123.

*Isolation of bone marrow cells and generation of bone marrow-derived dendritic cells.* Bone marrow-derived dendritic cells were generated according to previously published procedures by Bros et al..^[1]^ Briefly, bone marrow (BM) was obtained from femur and tibia of mice by flushing out using a 20 ml syringe and a 26 G needle and test medium (IMDM containing 5% FCS, 2 mM L-glutamine, 100 U/mL penicillin, 100 µg/mL streptomycin, 50 µM β-mercaptoethanol). Medium and supplements were purchased from Sigma Aldrich (United States) and Thermo Fisher Scientific (United States). FBS was purchased from PAN-Biotech (Germany). Erythrocytes were lysed with 1 mL Gey’s Red Cell Lysis buffer (155 mM NH_4_Cl, KHCO_3_, EDTA 100 µM, pH 7.4) for 1 min. at 4 °C and BM cells were washed with test medium and subsequently seeded in petri dishes (2x10^6^/dish) in 10 mL test medium supplemented with 10 ng/mL GM-CSF (BMDC medium). BMDC medium was replenished on days 3 and 6 of culture. BMDCs were differentiated from BM progenitor cells by culturing for 7 to 8 days and subsequently used for further *in vitro* uptake studies.

*Isolation of splenocytes.* Spleen cells were obtained as previously described ^[2]^. Briefly, spleens of mice were dissected and mechanically disrupted into single cells by grinding through a 40 µm cell strainer. Erythrocytes were lysed using Gey’s Red Cell Lysis buffer as described above and subsequently washed using test medium.

*MutuDC1940 uptake in vitro.* 100,000 cells per well were seeded in a 24-well plate and incubated at 37 °C, 5% CO_2_ overnight. 30 µg/mL nanocarriers were dispersed in medium (IMDM^+FBS^) and 250 µL of the resulting dispersions were added to each well in triplicates. After incubation (2 h at 37 °C), the cells were washed with 1 mL PBS per well to remove excess nanocarriers. 250 µL/well PBS with 5 mM EDTA and 10 mM HEPES was added to collect the cells. The plate was then kept at 37 °C for 5 min, before 250 µL/well medium was added and the samples were transferred into Eppendorff tubes. The media were removed by centrifugation (400 g, 5 min), and the cell pellet was resuspended in 1 mL PBS for flow cytometry measurements.

*BMDC and splenocyte uptake in vitro.* BMDCs (40,000 per well) were seeded in a 96-well flat bottom plate in BMDC medium and splenocytes (2x10^6^ per well) were seeded in a 48-well plate in test medium. Nanocarriers were added at a final concentration of 30 µg/ml. Cells were incubated overnight at 37 °C and 7.5% CO_2_. Subsequently, cells were harvested after incubation on ice for 20 min and stained for flow cytometry as described below.

*Cell staining.* Cells were washed with PBS and dead cells were stained using Live/Dead Aqua (1:1000 in PBS; Thermo Fisher Scientific) for 20 min at 4 °C. Cells were washed twice with PBS and Fc receptors were blocked with anti-CD16/CD32 (clone 2.4G2) for 15 min at 4 °C. BMDCs were subsequently stained with a fluorophore-conjugated antibody against CD11c (clone N418). Different cell types of splenocytes were differentiated via flow cytometry and therefore incubated with fluorophore-conjugated antibodies for 30 min: anti-CD11c (clone N418), anti-CD11b (clone M1/70), anti-CD172a (clone P84), anti-CD8α (clone 53-6.7), anti-I-A/I-E (MHCII, clone M5/114.15.2), anti-Siglec-H (clone 551), anti-CD19 (clone 6D5), anti-CD3ε (clone 145-2C11), anti-CD14 (clone Sa14-2), anti-NK1.1 (clone PK136), anti-Ly6G (clone 1A8), anti-XCR1 (ZET). Flow cytometric analyses were performed with the Attune NxT (Thermo Fisher Scientific) and analyzed with FlowJo software v10.9.0.

*In vivo biodistribution study.* Nanocarriers (500 µg in 200 µl PBS) were injected intravenously into tail veins of mice and organs (heart, lung, liver, spleen, kidney, and inguinal lymph nodes) were dissected and imaged via small animal fluorescence imaging (IVIS® SpectrumCT, Perkin Elmer). Single cell suspensions of spleens were prepared as described above.

*cLSM Experiment.* Confocal laser scanning microscopy (cLSM) was performed on an LSM SP5 STED Leica Laser Scanning Confocal Microscope (Leica, Germany), composed of an inverse fluorescence microscope DMI 6000CS with a multi-laser combination using a HC PL APO CS2 63x 12 water objective. Nanocarriers were excited with an argon excitation laser at 561 nm, and the plasma membrane stain CellMask^TM^ DeepRed (ThermoFisher) was excited using a HeNe excitation laser at 633 nm. In preparation for the experiment, 100 000 cells/well were seeded into 15 µ-slide 8 well glass bottom Ibidi slides. In the case of DC2.4, the slides were incubated overnight (37 °C, 5% CO_2_) before the nanocarriers were added at 30 µg/mL in 300 µL medium per well. For BMDCs, the nanocarriers were added to the cells directly without prior incubation of the cells in the Ibidi slides. After incubation (24 h, 37 °C), the samples were stained with CellMask^TM^ DeepRed plasma membrane stain to prepare for evaluation by confocal laser scanning microscopy (cLSM). For DC2.4, the staining was performed according to the supplier’s instructions: Shortly before analysis, the medium was removed from the well and 200 µL of an 1:1000 dilution of CellMask^TM^ DeepRed in PBS were added. After 5 min, the staining solution was removed, and 200 µL PBS were added before measuring. Since BMDCs do not adhere to the wells, we modified the protocol accordingly: 2 µL of a 1:10 dilution of CellMask^TM^ Deep Red were added to the well, and cLSM analysis was performed after 5 min.

*Statistical Analysis.* Statistical analyses were performed using GraphPad Prism version 10.3. All *in vitro* assays were performed with N = 3 and the *in vivo* biodistribution assays was performed with N = 4-5. To evaluate statistically significant differences between groups a One-Way ANOVA test (Brown-Forsythe and Welch ANOVA test) followed by a Dunnett's T3 multiple comparison post-hoc test was performed. *p* values < 0.05 were considered statistically significant.

**References**

[1] M. Bros, E. Montermann, A. Cholaszczynska, A. B. Reske-Kunz, *Int Immunopharmacol* **2016**, 35, 174.

[2] D. Passlick, K. Piradashvili, D. Bamberger, M. Y. Li, S. Jiang, D. Strand, P. R. Wich, K. Landfester, M. Bros, S. Grabbe, V. Mailänder, *J Control Release* **2018**, 289, 23.
